# Complex I Modulator BI4500 Reduces MASH by Limiting Oxidative Stress and Reprogramming Lipid Metabolism via AMPK in MCD Rats

**DOI:** 10.3390/antiox15010082

**Published:** 2026-01-08

**Authors:** Laura Giuseppina Di Pasqua, Sofia Lotti, Michelangelo Trucchi, Giuseppina Palladini, Anna Cleta Croce, Francesca Protopapa, Fausto Feletti, Stefan G. Kauschke, Peng Sun, Mariapia Vairetti, Andrea Ferrigno

**Affiliations:** 1 Unit of Cellular and Molecular Pharmacology and Toxicology, Department of Internal Medicine and Therapeutics, University of Pavia, 27100 Pavia, Italyandrea.ferrigno@unipv.it (A.F.); 2 Internal Medicine Fondazione IRCCS Policlinico San Matteo, 27100 Pavia, Italy; 3 Institute of Molecular Genetics, Italian National Research Council (CNR), 27100 Pavia, Italy; 4 Department of Biology and Biotechnology, University of Pavia, 27100 Pavia, Italy; 5 Department of Cardio-Renal Metabolic Diseases Research, Boehringer Ingelheim Pharma GmbH & Co. KG, 88397 Biberach an der Riss, Germany

**Keywords:** BI4500, MASH, AMPK, oxidative stress

## Abstract

Background: Metabolic-dysfunction-associated steatotic liver disease (MASLD) is a multifactorial liver disease in which mitochondrial dysfunction, oxidative stress, and inflammation play key roles in driving the progression toward metabolic dysfunction-associated steatohepatitis (MASH) and hepatocellular carcinoma (HCC). Dysfunctional mitochondria generate excess reactive oxygen species (ROS), impair antioxidant defenses, activate pro-inflammatory pathways and hepatic stellate cells, and perpetuate liver injury. Mitochondrial Complex I is a major ROS source, particularly under conditions of dysregulated energy metabolism. Since Complex I inhibition by metformin was shown to reduce ROS and activate the adenosine monophosphate-activated protein kinase (AMPK), this study aimed to evaluate whether a novel Complex I Modulator (CIM, BI4500) could attenuate oxidative stress, inflammation, and consequently reduce lipid accumulation and fibrosis in a methionine- and choline-deficient diet (MCD)-fed rat model of MASH. Methods: Rats were fed an MCD or an isocaloric control diet for six weeks. From week four, animals received daily oral treatment with CIM (10 mg/kg) or vehicle (Natrosol). At the endpoint, liver tissue was collected for histological, biochemical, and molecular analyses. Lipid droplet area, inflammatory infiltration, and collagen deposition were evaluated on tissue sections; total lipid content and oxidative stress markers were assessed in homogenates and isolated mitochondria. Molecular pathways related to oxidative stress, lipid metabolism, and fibrosis were assessed at protein and mRNA levels. Results: CIM treatment significantly reduced oxidative stress (ROS, lipid peroxidation, nitrogen species), promoting AMPK activation and metabolic reprogramming. This included increased expression of peroxisome proliferator-activated receptor alpha (PPAR-α) and its target genes, and decreased sterol regulatory element binding protein-1c (SREBP-1c)-driven lipogenesis. These changes halted fibrosis progression, as confirmed by Picro-Sirius Red staining and fibrosis markers. Conclusions: these findings indicate that Complex I modulation may represent a promising strategy to counteract MASLD progression toward MASH.

## 1. Introduction

About 30% of the worldwide population suffers from metabolic dysfunction-associated steatotic liver disease (MASLD), which is one of the leading causes of liver-related mortality all over the world [1,2]. The global rise of MASLD reflects the increasing incidence of metabolic syndrome that includes obesity, type 2 diabetes, dyslipidemia, and hypertension [3,4], with its prevalence reaching 60–75% in patients affected by these conditions [5,6]. MASLD firstly appears as simple steatosis, but it can progress to metabolic dysfunction-associated steatohepatitis (MASH), a more serious condition characterized by histologically proven lobular inflammation, hepatocyte ballooning, and fibrosis [2,7], potentially followed by cirrhosis and hepatocellular carcinoma (HCC) [8]. The resulting social and medical burden leads to a significant economic impact, prompting substantial investment in research on the pathogenesis and prevention of MASLD. Resmetirom, a selective thyroid hormone receptor β (THR-β) agonist, and Wegovy (semaglutide), a glucagon-like peptide 1 (GLP-1) agonist, have been recently approved as the first pharmacological agents specifically indicated for the treatment of MASH [7,9]; however, only two agents are unlikely to address the complex network of molecular mechanisms underlying the disease.

There is a broad consensus that inflammation and reactive oxygen species (ROS) play a key role in the transition from MASLD to liver carcinoma [10]. Currently available ROS scavengers are employed in MASLD to enhance endogenous antioxidant defenses—which include glutathione, vitamin E, associated antioxidant enzymes, and uncoupling proteins such as uncoupling protein 2 (UCP2) [11,12]—alongside a healthy lifestyle, insulin sensitizers, and gluconeogenesis inhibitors [13,14]. Nonetheless, a specific pharmacological target to address oxidative stress and effective treatment have yet to be identified.

ROS contribute both directly and indirectly to the overproduction of pro-inflammatory cytokines and the establishment of a chronic inflammatory response: decreasing the antioxidant function, activating hepatic stellate cells (HSC), and promoting the transition to fibrosis [10]. As mitochondria are the primary source of intracellular ROS, mitochondrial dysfunction plays a central role in the development of oxidative stress [14]. Mitochondrial β-oxidation is essential for energy production, but its defective functioning contributes to lipid accumulation and hepatic steatosis [15], triggering a vicious cycle. Lipid accumulation, in fact, promotes mitochondrial β-oxidation [16], induction of tricarboxylic acid cycle (TCA), and stimulation of oxidative phosphorylation (OXPHOS) [17] as an adaptive response to excess lipids. A consequence is an increase in reducing equivalents, flavin adenine dinucleotide, reduced and nicotinamide adenine dinucleotide (FADH_2_ and NADH), ROS, and oxidative stress [18]. These deleterious agents can target various cellular components, including membrane lipids, proteins, mitochondrial membranes, and mitochondrial DNA (mtDNA), and even respiratory chain complexes themselves, thereby impairing mitochondrial bioenergetics and promoting cell death, inflammation, and fibrosis [14,15,19]. The release of mtDNA in cytoplasm after ROS-caused mitochondrial disruption, in addition, promotes the activation of NLRP3 inflammasome and cytokine production, which in turn activate Kupffer cells, continuing to potentiate the hepatic inflammation [20]. ROS-activated Kupffer cells release TGF-β that triggers HSC activation, making them collagen-producing myofibroblastic cells and promoting fibrosis again. ROS also activate HSCs directly [14,21].

Complex I, the first of the five multi-subunit enzyme complexes in the mitochondrial respiratory chain, serves as the primary entry point for electrons derived from NADH to drive ATP synthesis. NADH is generated from multiple metabolic pathways, including glycolysis, with subsequent mitochondrial import via the pyruvate–tricarboxylic acid cycle, as well as the catabolism of amino acids and fatty acids through the acetyl-CoA pathway. This latter pathway also generates FADH_2_, which directly donates electrons to coenzyme Q within the respiratory chain. An imbalance between substrate supply to the respiratory complexes and the rate of ATP synthesis can lead to electron leakage, particularly at Complex I, resulting in the overproduction of ROS [22,23,24]. Especially, excessive ROS leakage from Complex I is believed to contribute to the pathogenesis of both MASLD and its progression to MASH [25].

Cameron and colleagues demonstrated that metformin, an insulin sensitizer, directly targets mitochondrial Complex I, thereby opening a promising avenue for further research [26].

Metformin-induced inhibition of Complex I reduces the [ATP]/[ADP] and [ATP]/[AMP] ratios, thereby activating AMP-activated protein kinase (AMPK), a central regulator of lipid metabolism [27]. AMPK activation reduces lipogenesis through the phosphorylation and inhibition of acetyl-CoA carboxylase 1 (ACC1) and ACC2, and increases hepatic fatty acid oxidation [28]. Additionally, AMPK counteracts glucagon signaling by limiting cyclic AMP accumulation and directly prevents gluconeogenesis via inhibition of fructose 1,6-bisphosphatase; however, these latter effects are not observed at metformin concentrations typically achieved in clinical settings [27,29].

The finding that metformin—used in MASLD management for its ability to reduce hepatocyte ballooning—also modulates ROS production at Complex I strengthens the rationale for considering Complex I as a potential pharmacological target. However, metformin exerts pleiotropic effects, and whether selective modulation of Complex I–derived ROS is sufficient to ameliorate MASH remains unclear.

This study aims to investigate whether the selective modulation of mitochondrial Complex I, by a novel Complex I Modulator (CIM, BI4500), could improve MASH in methionine and choline-deficient diet (MCD)-fed rats. BI4500 is a novel indolinone compound (C_20_H_27_N_3_O_3_, ≥99.5% purity) developed by Boehringer Ingelheim, selectively suppresses superoxide production from the complex I ubiquinone-binding site (I_Q_) of the reverse electron transport (RET), without acting as a general complex I inhibitor or direct ROS scavenger. Unlike classical site I_Q_ inhibitors like rotenone, CIM inhibits I_Q_ ROS release, preserving complex I–dependent mitochondrial respiration [30]. We hypothesized that selective modulation of mitochondrial Complex I–derived ROS by CIM could attenuate liver injury and metabolic dysfunction in MASH. Therefore, to test this hypothesis, we first investigated the effects of CIM on hepatic oxidative stress and inflammation. Secondly, we moved our attention to lipid metabolism and early fibrotic changes in MCD diet-fed rats, as well as to explore the involvement of AMPK signaling.

## 2. Materials and Methods

### 2.1. Animal Model and Experimental Procedures

The animal model used in this study was approved by the Italian Ministry of Health and the Animal Care Committee of the University of Pavia (Authorization number 163/2020—5 March 2020), and this study was conducted following the ARRIVE guidelines. Five-week-old male Wistar rats (Charles River Laboratories, Calco, LC, Italy) were housed under standard conditions and, after a one-week quarantine period, were fed either a methionine- and choline-deficient (MCD) diet or an isocaloric control (CTRL) diet (Laboratorio Dottori Piccioni, Gessate, MI, Italy) for a total duration of 6 weeks. The control diet is identical to the MCD diet in terms of caloric content, but it is supplemented with L-methionine and L-choline (the two diets’ composition formulations are reported in Appendix A, Table A1).

Starting from week 4, animals received daily oral administration (via gavage) of 10 mg/kg of the Complex I Modulator (CIM; groups: CTRL + CIM (n = 6) and MCD + CIM (n = 15)) or vehicle (0.5% Natrosol; groups: CTRL + NA (n = 6) and MCD + NA (n = 15)). The treatment duration and dosage were defined by the sponsor, Boehringer Ingelheim Pharma GmbH & Co. KG (Biberach an der Riss, Germany). At the end of the experimental protocol, rats were anesthetized with an intraperitoneal injection of pentobarbital (40 mg/kg) in a non-fasting state, for blood collection from the inferior vena cava and subsequent plasma separation. Hepatic tissue samples from the left, median, and right lobes were harvested and either snap-frozen in liquid nitrogen for biochemical analysis or fixed in formalin for histological processing and paraplast embedding. In a subset of MCD-fed rats (MCD + NA, n = 5; MCD + CIM, n = 5), the hepatic left lobe was used for fresh mitochondria isolation.

### 2.2. Materials

Complex I Modulator (CIM, BI4500) was purchased from Boehringer Ingelheim Pharma GmbH & Co. KG. CIM is an indolinone (C_20_H_27_N_3_O_3_) with greater than 99.5% purity. All chemicals, when not specified, were obtained from Merck/Sigma-Aldrich (Milano, Italy).

### 2.3. Plasma Sample Preparation for Enzyme and Biochemical Parameter Evaluation

Plasma samples were obtained from blood collected from the inferior vena cava into Na_2_EDTA tubes, followed by centrifugation at 1520× *g* for 15 min at room temperature. Aliquots were then sent to MyLav–Laboratorio La Vallonea (Passirana di Rho, MI, Italy) for the assessment of alanine aminotransferase (ALT), aspartate transaminase (AST), alkaline phosphatase (ALP), bilirubin, cholesterol, and triglyceride levels.

Lactate dehydrogenase (LDH) release was measured spectrophotometrically at 340 nm, by assessing enzymatic activity in the presence of saturating concentrations of NADH and pyruvate, as previously described [31].

### 2.4. Analysis of Oxidative Stress and Reactive Oxygen/Nitrogen Species

Lipid peroxidation in liver homogenates was assessed by measuring thiobarbituric acid reactive substances (TBARS), following the method described by Esterbauer and Cheeseman [32]. Malondialdehyde (MDA) served as the reference standard for TBARS quantification.

Hepatic ROS production was evaluated using the dihydro-dichlorofluorescein diacetate (DCFH-DA) assay, which relies on the oxidation of DCFH to fluorescent dichlorofluorescein in the presence of ROS, as previously reported [33].

Hepatic content of nitrate + nitrite (NOx) was quantified using a commercial assay kit (Cayman Chemical, Ann Arbor, MI, USA). Prior to analysis, samples were filtered through a 30-kDa molecular weight cut-off filter to remove proteins. The filtrates were then mixed in equal volume with Griess reagent, incubated for 10 min at room temperature under reduced light conditions, and absorbance was measured at 540 nm [34].

### 2.5. Mitochondria Isolation, ROS, and Respiratory Control Ratio (RCR) Evaluation

In a subset of MCD-fed rats, the hepatic left lobe was used for fresh mitochondria isolation, according to the Lehninger et al. method of differential centrifugation [35]. Briefly, the left hepatic lobe was rinsed in ice-cold homogenization buffer (0.25 M sucrose, 1 mM EDTA, 5 mM HEPES, pH 7.2), weighed, minced, and homogenized in a Potter–Elvehjem Teflon/glass homogenizer (Sartorius, Göttingen, Germany) with a volume of buffer twice the tissue weight. The homogenate was filtered through double gauze and centrifuged at 500× *g* for 10 min. The supernatant underwent two sequential centrifugations (9400× *g* and 10,000× *g*, 10 min each), with the final pellet resuspended in an EDTA-free buffer (0.25 M sucrose, 5 mM HEPES) [10]. Individual mitochondrial preparations were obtained (MCD + NA, n = 5; MCD + CIM, n = 5), and protein content was determined using the Lowry method [36]. Isolated mitochondria were kept on ice for immediate use in respiratory control ratio (RCR) and ROS assays.

Mitochondrial oxygen consumption was measured at 25 °C using a Clark-type electrode in a sealed chamber. As previously described, isolated mitochondria (1 mg/mL) were added to 2 mL of respiration buffer (5 mM Mg^2+^, 0.5 mM EGTA, 70 mM sucrose, 220 mM mannitol, 10 mM KH_2_PO_4_, and 10 mM Tris-HCl, pH 7.4). After stabilization, respiration was initiated with 10 mM succinate and 1 μM rotenone, and oxidative phosphorylation was triggered by 0.5 mM ADP. The respiratory control ratio (RCR) was calculated as the ratio between State 3 (ADP-stimulated respiration) and State 4 (resting respiration after ADP consumption) [37].

Mitochondrial ROS production was evaluated by incubating freshly isolated mitochondria (1 mg/mL) with 5 µM DCFH-DA for 10 min in the dark. After centrifugation (660× *g*, 10 min, RT), the pellet was resuspended in respiration buffer. Aliquots (2 mL) were transferred into a UV quartz cuvette, and ROS generation was stimulated with 10 mM succinate and 1 μM rotenone, followed by 0.5 mM ADP. Fluorescence intensity (Ex 493 nm/Em 520 nm) was recorded for 10 min using an LS 50 B spectrometer (Perkin Elmer Inc., Waltham, MA, USA). Results are expressed as State 3 DCFH fluorescence % increase [10].

### 2.6. ATP Content and NAD(P)H Bound/Free Ratio Evaluation

ATP content and ATP/ADP ratio in liver samples were determined using a luciferin-luciferase bioluminescence assay kit (Perkin Elmer Inc., Waltham, MA, USA) and normalized to protein concentration (nmol/mg protein). Luminescence signals were recorded with a Wallac Victor^2^ multilabel plate reader (Perkin Elmer Inc., Waltham, MA, USA) using white 96-well microplates.

Nicotinamide adenine dinucleotide phosphate, reduced form (NAD(P)H) bound/free ratio was calculated as previously described [10,38]. Autofluorescence spectra were acquired from unfixed liver cryosections using a microspectrograph (Leitz, Wetzlar, Germany) under epi-illumination with a 100 W/Hg lamp (Osram, Berlin, Germany) and excitation at 366 nm (interference filter, FWHM = 10 nm). Emission was collected through a 50/50 dichroic mirror and a 390 nm long-pass filter, using a 40× objective (n.a. 0.75), and transferred via optical fiber to a multichannel analyzer (Hamamatsu PMA-12, Arese, Italy). Spectra (390–700 nm) were normalized (100 a.u. at the peak) and analyzed using PeakFit software (PeakFit version 4; SPSS Science, Chicago, IL, USA) with the Marquardt algorithm. Endogenous fluorophores were resolved using half-Gaussian Modified Gaussian (GMG) functions based on peak position (λ) and FWHM. The contribution of each fluorophore was calculated from its relative spectral area. NAD(P)H bound/free ratios were derived from the spectral bands at 444 nm (FWHM = 105 nm) and 463 nm (FWHM = 115 nm). Fitting quality was evaluated by residuals and r^2^ values.

### 2.7. Hepatic Total Lipid Content Assay

Total lipid extraction from frozen liver samples (50–70 mg) was performed according to Lyn-Cook et al. [39], as previously described [40]. Briefly, tissues were homogenized in 200 µL of water, and lipids were extracted with 1 mL of chloroform-methanol (2:1, *v*/*v*), followed by 1 h incubation at room temperature with intermittent agitation. After centrifugation (1520× *g*, 5 min, RT), the lower organic phase was collected, dried under nitrogen, and resuspended in 100 µL of absolute ethanol. For quantification, 12.5 µL of lipid extract was mixed with 475 µL of phosphate-buffered saline and 12.5 µL of Nile Red solution (1 mg/mL in DMSO) in a UV quartz cuvette. Fluorescence intensity (Ex 485 nm/Em 572 nm) was measured using an LS 50 B fluorescence spectrometer (Perkin Elmer Inc., Waltham, MA, USA). Lipid content was expressed as fluorescence/mg proteins.

### 2.8. Hepatic Tissue Histology and Staining

Liver samples from the left lobe were fixed in 2% paraformaldehyde in 0.1 M phosphate buffer (pH 7.4) for 24 h, then processed and embedded in Paraplast wax. Sections (8 μm) were stained with Hematoxylin–Eosin (Sakura Finetek, Mestre, Italy) or Sirius Red (Direct Red 80, Sigma-Aldrich, Milan, Italy). The stained slides were examined under a light microscope (Nikon Eclipse E800; Nikon Instruments s.p.a, Campi Bisenzio, FI, Italy). Lipid droplet area and fibrosis were quantified using ImageJ (ij153-win-java8 version), while inflammatory cell infiltration was assessed using a semi-quantitative histopathology score, as previously described [41].

### 2.9. Nuclear Fraction Extraction from Frozen Tissue

Nuclear extracts from frozen rat livers were obtained as follows. Two buffers were prepared prior to extraction: a hypotonic buffer (10 mM HEPES, 10 mM KCl, 0.1 mM EDTA, pH 7.9) and a hypertonic buffer (20 mM HEPES, 400 mM NaCl, 0.1 mM EDTA, pH 7.9), both supplemented at the time of use with 1 mM DTT and a protease inhibitor cocktail (10 μL/mL). Tissues were homogenized in 1 mL of supplemented hypotonic buffer using a Potter homogenizer, incubated on ice for 10 min, then treated with 0.05% Nonidet P-40 and incubated for an additional 5 min with brief agitation. Then, samples were centrifuged at 400× *g* for 2 min at 4 °C to separate the cytoplasmic fraction (supernatant), which was stored in liquid nitrogen. The nuclear pellet was washed three times with hypotonic buffer containing 0.1% Nonidet P-40, then resuspended in 100 μL of hypertonic buffer, and vortexed on ice every 5 min for 30 min. After centrifugation at 15,000× *g* for 10 min at 4 °C, the nuclear extracts (supernatant) were collected and stored. Protein concentration in both cytoplasmic and nuclear fractions was determined by Lowry assay on 4 μL of each sample [36].

### 2.10. Western Blot Analysis

Liver tissue samples were homogenized in ice-cold lysis buffer supplemented with protease inhibitors, followed by centrifugation at 15,000× *g* for 10 min. Equal amounts of protein from liver lysates or nuclear extracts were resolved by SDS-PAGE on 7.5–10 or 12% acrylamide gels and subsequently transferred onto PVDF membranes. Non-specific binding sites were blocked with 5% bovine serum albumin (BSA) in TBS-T (20 mM Tris-HCl, 500 mM NaCl, 0.1% Tween-20, pH 7.5) for 2 h at 4 °C. Membranes were then incubated overnight at 4 °C with primary antibodies under gentle agitation at appropriate dilution. Antibody dilutions and suppliers are listed in Appendix A, Table A2.

After incubation with primary antibodies, membranes were washed in TBS-T and incubated with horseradish peroxidase (HRP)-conjugated secondary antibodies (anti-rabbit or anti-mouse) at the appropriate dilutions. Immunostaining was revealed with BIO-RAD Chemidoc XRS+ visualized using the ECL Clarity BIO-RAD (Segrate, MI, Italy). Band intensities were quantified using BIO-RAD Image Lab™ Software 6.0.1 (Segrate, MI, Italy).

### 2.11. RT-PCR Analysis

Total RNA was extracted from a median lobe of frozen liver tissue using TRI Reagent (Sigma-Aldrich, Milano, Italy), following the Chomczynski method [42]. RNA concentration and purity were assessed by measuring absorbance at 260 nm and 280 nm using a T92^+^ UV spectrophotometer (PG Instruments Ltd., Lutterworth, UK) and calculating the 260/280 nm ratio. cDNA synthesis was performed using the iScript Supermix (BIO-RAD, Segrate, MI, Italy). Quantitative PCR (qPCR) was carried out on a CFX96™ Real-Time System (BIO-RAD) using 10 μL of SsoAdvanced™ SYBR^®^ Green Supermix, 1 μL of forward/reverse primer mix (10 pmol/μL), and 2 μL of cDNA (2.5 ng/μL), in a final volume of 20 μL per well. Amplification was performed in two-step cycles (95–60 °C) for 40 cycles according to the manufacturer’s protocol. Each sample was run in triplicate. Amplification efficiencies for target and reference genes were determined via standard curves over a cDNA dilution range (5–0.625 ng/μL), ranging from 97.2% to 106.1%. The expression levels of the reference genes (*Ubc*, *Gapdh*, and *Rps9*) remained stable across experimental conditions. Primer amplicon sequences and unique assay IDs (PrimePCR, BIO-RAD) are reported in Appendix A, Table A3, in accordance with MIQE guidelines [43]. Gene expression was calculated using the ΔCt method, and group comparisons were performed using the ΔΔCt method.

### 2.12. Gelatin Zymography

Protein extraction from snap-frozen tissue samples and gelatin zymography were carried out as previously described [44]. To assess matrix metalloproteinase (MMP) activity, solubilized proteins were separated by SDS-PAGE on gels containing 1 mg/mL gelatin under non-reducing conditions. Following electrophoresis, gels were incubated for 18 h at 37 °C in a specific renaturation and development buffer. Gelatinolytic activity was visualized by staining with Coomassie Brilliant Blue, revealing clear bands corresponding to zones of substrate degradation. Zymograms were analyzed using a GS-900 densitometer (BIO-RAD).

### 2.13. Statistical Analysis

Statistical analyses were conducted using R (version 4.1.0) and RStudio (version 2022.02.3, Build 492). Sample size was estimated a priori using power analysis with R (pwr package), considering the number of experimental groups, effect sizes from previous experiments in our laboratory and/or literature, and potential unforeseen events during the 6-week treatment period. An effect size of 0.7, 80% power, and a significance level of *p* = 0.05 were assumed. Analyses were performed using base R functions (stats package) and the rstatix package (version 0.7.2) for post hoc and non-parametric analyses. Post hoc tests were selected based on the data distribution and variance homogeneity. Specifically, after a significant one-way ANOVA, Tukey’s test was used when assumptions of normality and homogeneity of variances were met, whereas Dunn’s test was applied for non-normal distributions. When homogeneity of variance was violated, the Games–Howell test was employed as a robust alternative. For two-group comparisons, Student’s *t*-test was used for normally distributed data, and Wilcoxon’s rank-sum test for non-normal distributions. Normality and variance homogeneity were assessed using Shapiro–Wilk and Levene’s tests, respectively. No missing values were present in the datasets. Results are presented as mean ± standard error of the mean (S.E.), with a significance threshold set at *p*  <  0.05.

## 3. Results

In this study, rats were fed either MCD or an isocaloric control diet (CTRL) for 6 weeks and, starting from the fourth week, animals were orally administered 10 mg/kg/day of CIM or vehicle (Natrosol, NA). The MCD diet, rich in sucrose and fats but lacking methionine and choline, impairs lipid oxidation and VLDL synthesis, leading to hepatic lipid accumulation, oxidative stress, and inflammation. Within 8 weeks, it induces steatohepatitis, characterized by weight loss, liver atrophy, and macrophage activation. The isocaloric control diet has identical composition, but it is supplemented with L-methionine and L-choline. This model is widely used to study lipotoxicity, inflammation, fibrosis, and HCC [8]. At the time of sacrifice, rats were weighed, and basic phenotyping information on body weight and liver weight for all groups is reported in Table 1.

### 3.1. Model Characterization and Liver Injury Evaluation: Role of CIM

Liver injury was assessed by measuring plasma levels of transaminases (AST, ALT), alkaline phosphatase (ALP), lactate dehydrogenase (LDH), total bilirubin (as a marker of cholangiocyte stress), and cholesterol and triglycerides (as indicators of lipid metabolism). As expected, MCD rats showed a significant increase in AST, ALT, and total bilirubin, along with reduced cholesterol and triglycerides, compared with CTRL rats. CIM treatment in CTRL rats significantly reduced AST, ALT, LDH, and triglycerides, while no significant changes were observed between treated and untreated MCD rats (Table 2).

### 3.2. Role of CIM in Hepatic Oxidative Stress, Mitochondrial Dysfunction, and Energy Balance

**Figure 1:**
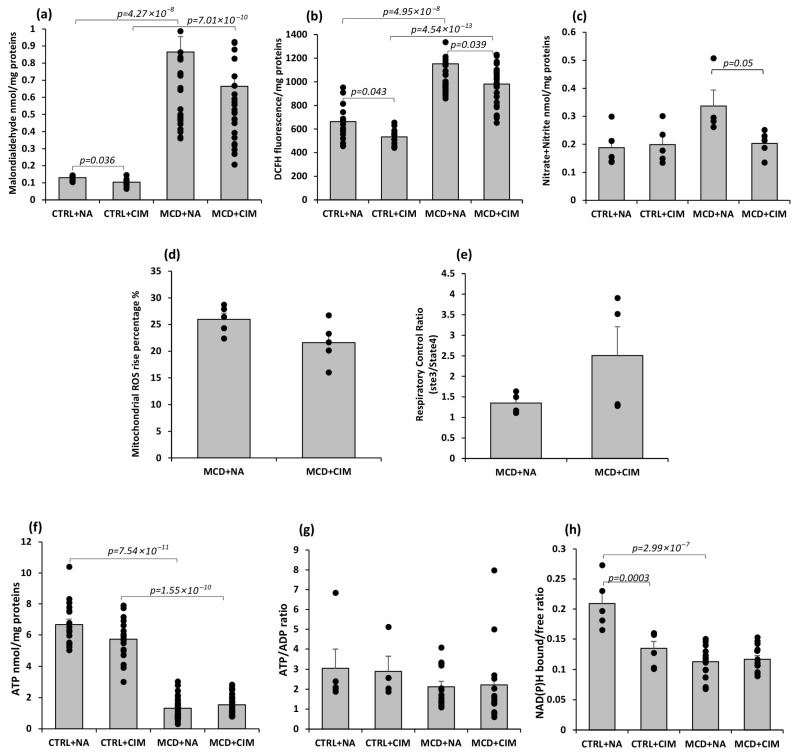
Oxidative stress markers, mitochondrial ROS and RCR, energy balance, and mitochondrial efficiency evaluation in 6-week control or MCD-fed rats following CIM or vehicle administration. Rats were fed a control (CTRL) diet for 6 weeks and, starting from week 4, received daily oral administration of either CIM (10 mg/kg/day; CTRL + CIM, n = 6) or vehicle (CTRL + NA, n = 6). The same treatment regimen was applied to rats fed an MCD diet, resulting in two groups: MCD + CIM (n = 15) and MCD + NA (n = 15). (**a**) TBARS evaluation was assessed spectrophotometrically at 535 nm using a T92^+^ UV spectrophotometer in hepatic tissue. MDA levels decreased significantly in CTRL rats treated with CIM. A similar, non-significant trend was observed in CIM-treated MCD rats. For each animal, the TBARS assay was performed in triplicate; (**b**) ROS levels in hepatic tissue were measured by recording fluorescence intensity (Ex 493 nm/Em 520 nm) using an LS 50 B spectrometer (Perkin Elmer Inc., Waltham, MA, USA). A significant reduction in ROS production was recorded in both CIM-treated CTRL and MCD rats. For each animal, the ROS were assayed in triplicate; (**c**) reactive nitrogen species (nitrate + nitrite) production in hepatic tissue was acquired following the kit instructions (Cayman Chemical). This assay was conducted on a randomly selected sample of N = 5 per group. CIM administration reduced nitrogen species in MCD rats in a significant way. (**d**) ROS evaluation in hepatic mitochondria from only MCD rats (MCD + CIM, n = 5) or vehicle (MCD + NA, n = 5) showed a reduction following CIM administration; (**e**) RCR in hepatic mitochondria was assessed using a Clark-type electrode in a sealed chamber. A recovery in RCR was found in CIM-treated MCD rats; (**f**) ATP evaluation in hepatic tissue was determined using a luciferin-luciferase bioluminescence assay kit (Perkin Elmer Inc.), and signals were recorded with a Perkin Elmer Wallac Victor2 multilabel plate reader. For each animal, the ATP detection was assayed in triplicate. A significant reduction was found in ATP levels in both MCD groups. No changes were observed after CIM administration. (**g**) ATP/ADP ratio: no significant differences were found among the experimental groups; (**h**) NAD(P)H bound/free ratio in hepatic tissue was analyzed, recording autofluorescence spectra from unfixed liver cryosections; for detailed information on the equipment, refer to the Materials and Methods section. NAD(P)H bound/free ratio decreased in CIM-treated control rats compared with vehicle-treated controls. The results are expressed as mean ± S.E of 15 biological replicates for MCD groups and 6 biological replicates for CTRL groups. CTRL = isocaloric control diet; MCD = methionine and choline-deficient diet; NA = Natrosol (vehicle); CIM = Complex I Modulator.

Oxidative stress levels in hepatic tissue were assessed by measuring lipid peroxidation, ROS production, and nitrate + nitrite (NOx) release. Lipid peroxidation was evaluated by quantifying malondialdehyde (MDA) [45]. As expected, MCD groups showed significantly higher MDA levels than their respective controls. CIM treatment significantly reduced lipid peroxidation in CTRL rats, with a similar, though non-significant, trend observed in MCD rats (Figure 1a). ROS were evaluated using the fluorescent probe dihydro-dichlorofluorescein (DCFH). The MCD diet induced a significant increase in ROS production. CIM treatment resulted in a significant reduction in ROS levels in both CTRL and MCD (Figure 1b). CIM also elicited a significant decrease in NOx levels, but only in MCD rats (Figure 1c). Collectively, these data suggest that daily administration of CIM at a dose of 10 mg/kg reduces oxidative stress induced by hypercaloric (CTRL or MCD) diet administration.

To assess CIM’s effect on mitochondrial function, ROS production and respiratory control ratio (RCR) were measured in freshly isolated hepatic mitochondria from MCD rats. ROS levels, expressed as the percent increase from State 4 to State 3 respiration, were reduced by CIM treatment, with a trend toward significance (*p* = 0.07) compared with untreated mitochondria (Figure 1d). Mitochondrial oxygen consumption was also measured to assess mitochondrial function. The respiratory control ratio (RCR)—the ratio of oxygen consumption in active (State 3) versus resting (State 4) respiration—reflects the coupling efficiency of oxidative phosphorylation [46]. An increased RCR in hepatic mitochondria from CIM-treated MCD rats suggests partial recovery of mitochondrial function compared with untreated animals (Figure 1e).

To assess cellular energy status, hepatic ATP content, ATP/ADP ratio, and NAD(P)H bound/free ratio were measured in 6-week CTRL and MCD rats, with or without CIM treatment. As expected, ATP content was significantly reduced in both MCD groups. CIM had no significant effect, although a slight reduction was observed in treated CTRL rats, possibly reflecting a limited inhibition of mitochondrial respiration (Figure 1f). No significant differences were found in the ATP/ADP ratio among the experimental groups (Figure 1g). The ratio between bound and free NAD(P)H is a well-established indicator of mitochondrial functional status. Bound NAD(P)H reflects interaction with mitochondrial complex I and is associated with aerobic metabolism, whereas free NAD(P)H reflects cytosolic anaerobic pathways [47,48,49]. A decrease in the bound/free NAD(P)H ratio is generally interpreted as a sign of reduced mitochondrial efficiency, although this interpretation may vary depending on the metabolic context [38]. In CIM-treated control rats, the NAD(P)H bound/free ratio was significantly lowered compared with vehicle-treated controls. This finding may reflect the partial inhibitory effect of CIM on mitochondrial functionality. A significant decrease in this ratio was also observed in both MCD groups compared with the untreated control group, consistent with severe mitochondrial dysfunction and oxidative stress (Figure 1h).

### 3.3. CIM Promotes Mitochondrial Recovery and Ameliorates Antioxidant Defenses

The potential role of CIM in mitochondrial recovery and antioxidant defenses was assessed by measuring protein or mRNA levels of MIC-19, *Idh2*, *Ucp2*, *Nfe2l2*, and *Nqo1*.

**Figure 2:**
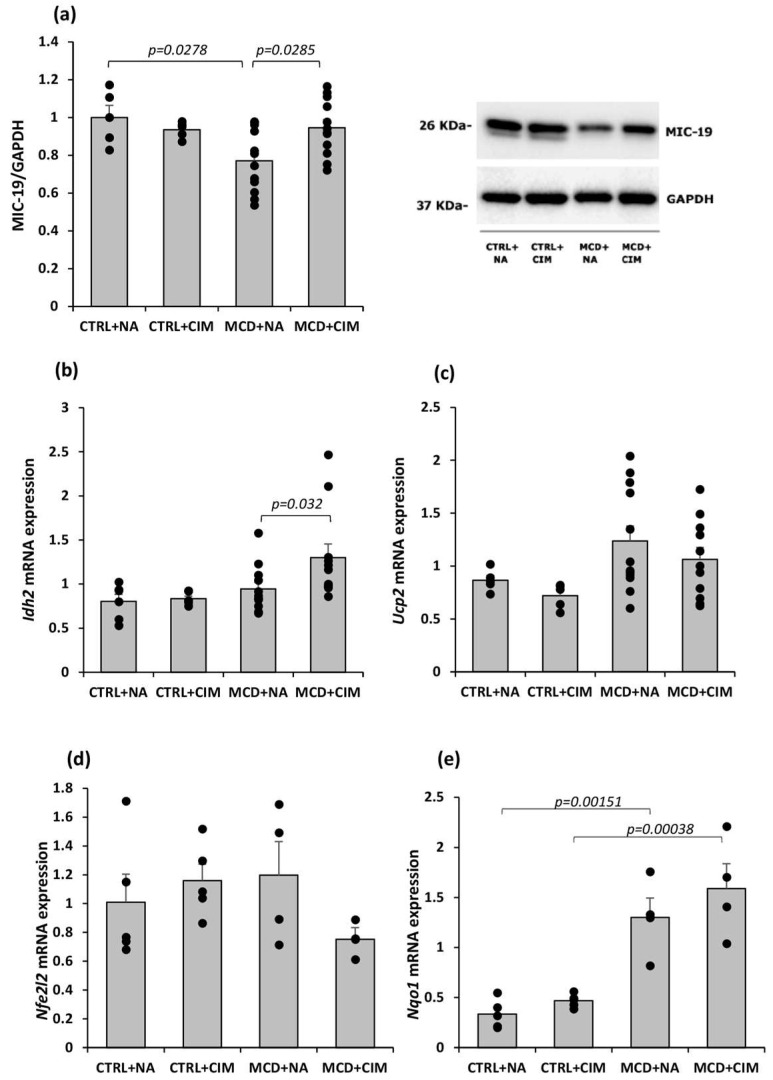
MIC-19 protein expression and antioxidant defense (*Idh2*, *Ucp2*, *Nfe2l2*, and *Nqo1*) mRNA expression evaluation by Western blot and RT-PCR in 6-week control or MCD-fed rats following CIM or vehicle administration. Rats were fed a control (CTRL) diet for 6 weeks and, starting from week 4, received daily oral administration of either CIM (10 mg/kg/day; CTRL + CIM, n = 6) or vehicle (CTRL + NA, n = 6). The same treatment regimen was applied to rats fed an MCD diet, resulting in two groups: MCD + CIM (n = 15) and MCD + NA (n = 15). (**a**) Western blot analysis of MIC-19 revealed that CIM significantly restored the cristae integrity in MCD rats to those observed in controls; (**b**) *Idh2* mRNA expression evaluation showed a significant increase in CIM-treated MCD rats; (**c**) *Ucp2* mRNA expression levels showed no changes among the four experimental groups; (**d**) *Nfe2l2* mRNA expression showed the same trend of *Ucp2*; (**e**) *Nqo1* mRNA expression was significantly upregulated in both MCD groups. The results are expressed as mean ± S.E. of 15 biological replicates for MCD groups and 6 biological replicates for CTRL groups. Samples were loaded onto three separate polyacrylamide gels, while each sample was analyzed in triplicate both for target genes and housekeeping genes in RT-PCR analysis. CTRL = isocaloric control diet; MCD = methionine and choline-deficient diet; NA = Natrosol (vehicle); CIM = Complex I Modulator.

MIC-19 is a key component of the mitochondrial contact site and cristae organizing system (MICOS); its role is maintaining the contact between the endoplasmic reticulum (ER) and mitochondria [50]. MICOS plays a critical role in mitochondrial homeostasis, and its disruption leads to oxidative stress and mitochondrial dysfunction [51,52]. Western blot analysis revealed a marked reduction in MIC-19 protein levels in the livers of MCD rats, indicating a disruption of mitochondrial integrity. Notably, CIM significantly counteracted this reduction, restoring MIC-19 levels to values comparable to those observed in the control groups (Figure 2a).

Isocitrate dehydrogenase 2 (IDH2) is a mitochondrial enzyme that catalyzes the decarboxylation of isocitrate to α-ketoglutarate. During this reaction, IDH2 reduces NADP^+^ to NAD(P)H, which is essential for mitochondrial antioxidant systems involving glutathione (GSH), such as glutathione peroxidase and glutathione reductase [53,54,55]. Moreover, reduced IDH2 activity is closely associated with lipid accumulation and hepatic dysfunction in both in vitro and in vivo models of steatosis [53]. Our results show a significant increase in *Idh2* mRNA expression in MCD rats treated with CIM, compared with untreated ones (Figure 2b).

UCP2 reduces mitochondrial ROS production by mildly lowering the mitochondrial membrane potential (ΔΨm), which relieves the backpressure on the electron transport chain, enhances electron flow, and reduces the likelihood of electron leakage to oxygen and subsequent ROS generation [56,57,58]. Under physiological conditions, UCP2 expression is low or even undetectable in normal liver cells; however, it is markedly upregulated in the context of MASH. While this upregulation may serve to limit ROS production, it can also reduce the efficiency of the mitochondrial respiratory chain, potentially affecting energy homeostasis and impairing the liver’s ability to meet acute energy demands [11]. As expected, the graph showed detectable levels of *Ucp2* mRNA expression in all groups. No significant changes were observed in the CTRL or MCD groups treated with CIM compared with their respective untreated controls. However, a trend to reduction was observed in the CIM-treated groups, suggesting that, since CIM directly reduces ROS levels, it might initiate a downregulation of *Ucp2* expression (Figure 2c).

One of the key genes involved in the regulation of antioxidant defenses is *Nfe2l2*, which encodes the nuclear transcription factor erythroid 2–related factor 2 (NRF2). NRF2 positively regulates a broad range of genes involved in protection against oxidative and electrophilic stress and, in rodents, also modulates hepatic lipid metabolism by repressing genes that promote hepatic steatosis [59]. In response to oxidative stress, NRF2 translocates from the cytoplasm to the nucleus, where it activates the transcription of various antioxidant and cytoprotective genes, including NAD(P)H dehydrogenase quinone 1 (NQO1) [60]. No significant changes in *Nfe2l2* mRNA expression were detected across the experimental groups, although a non-significant reduction was observed in the MCD group treated with CIM, likely reflecting the decreased oxidative stress markers observed following CIM treatment (Figure 2d). In contrast, *Nqo1* mRNA expression was significantly increased in both MCD groups compared with their respective controls, with CIM treatment in MCD rats causing a slight further increase in its expression (Figure 2e).

### 3.4. CIM Reduces Fat Accumulation and Modulates Lipid Metabolism Through AMPK Signaling Pathway

**Figure 3:**
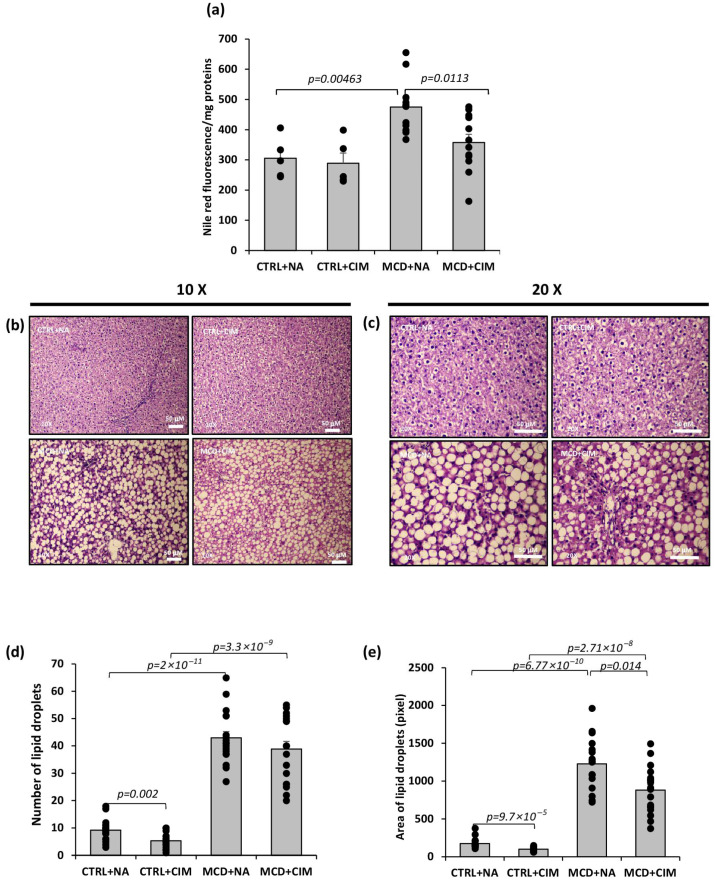
Total lipid content, number, and area of lipid droplets were evaluated in hepatic lipid extracts or slides from 6-week control or MCD-fed rats administered with CIM or vehicle. Rats were fed a control (CTRL) diet for 6 weeks and, starting from week 4, received daily oral administration of either CIM (10 mg/kg/day; CTRL + CIM, n = 6) or vehicle (CTRL + NA, n = 6). The same treatment regimen was applied to rats fed an MCD diet, resulting in two groups: MCD + CIM (n = 15) and MCD + NA (n = 15). (**a**) Total lipid content was assessed by measuring Nile red fluorescence intensity (Ex 485 nm/Em 572 nm) using an LS 50 B fluorescence spectrometer (Perkin Elmer Inc., Waltham, MA, USA). Lipid content was expressed as fluorescence/mg proteins, and a significant reduction was recorded in MCD rats treated with CIM; (**b**,**c**) Hematoxylin and Eosin-stained liver sections. Panels at 10× (**b**) and 20× (**c**) magnification are shown (scale bar: 50 μM). Slides were examined under a light microscope (Nikon Eclipse E800); (**d**,**e**) number and area, respectively, of lipid droplets in hepatic tissue quantified by ImageJ software. The analysis showed a significant decrease in both the number and the area of lipid droplets in CTRL rats treated with CIM; the same trend, although not significant, was shown only for the number in MCD rats. The results are expressed as mean ± S.E. of 15 biological replicates for MCD groups and 6 biological replicates for CTRL groups. Each sample was analyzed in duplicate for Nile red assay, while 3 different fields of 4 different biological replicates for each group were considered for ImageJ analysis. CTRL = isocaloric control diet; MCD = methionine and choline-deficient diet; NA = Natrosol (vehicle); CIM = Complex I Modulator.

Total lipid content was evaluated on liver extracts using the fluorescent dye Nile Red. MCD-fed rats displayed a significant increase in total lipids compared with their respective controls. CIM administration markedly reduced lipid accumulation in MCD rats, substantially attenuating MCD-induced alterations and partially normalizing the phenotype toward control levels (Figure 3a). To assess CIM’s effect on hepatic steatosis, liver slices from all groups were stained with hematoxylin and eosin to evaluate tissue architecture and lipid droplets. Control rats showed a healthy liver structure with some lipid droplets and sinusoidal dilation, both of which were reduced by CIM. MCD-fed rats exhibited hepatocyte ballooning, extensive macrovesicular steatosis, and leukocyte infiltration (Figure 3b,c). Quantification confirmed increased lipid droplet number in MCD groups versus controls. CIM significantly decreased droplet number in control rats and showed a non-significant reduction in MCD rats. Droplet area was larger in MCD rats but significantly reduced by CIM in both control and MCD groups (Figure 3d,e).

AMPK is a key regulator of hepatic lipid metabolism. AMPK activation inhibits lipogenesis by downregulating key enzymes and transcription factors such as ACC1, fatty acid synthase (FAS), and sterol regulatory element binding protein-1c (SREBP-1c). Concurrently, AMPK promotes fatty acid oxidation and lipid catabolism through the upregulation of downstream effectors, including peroxisome proliferator-activated receptor gamma coactivator 1-alpha (PGC1α), and carnitine palmitoyltransferase 1 (CPT1) [61].

**Figure 4:**
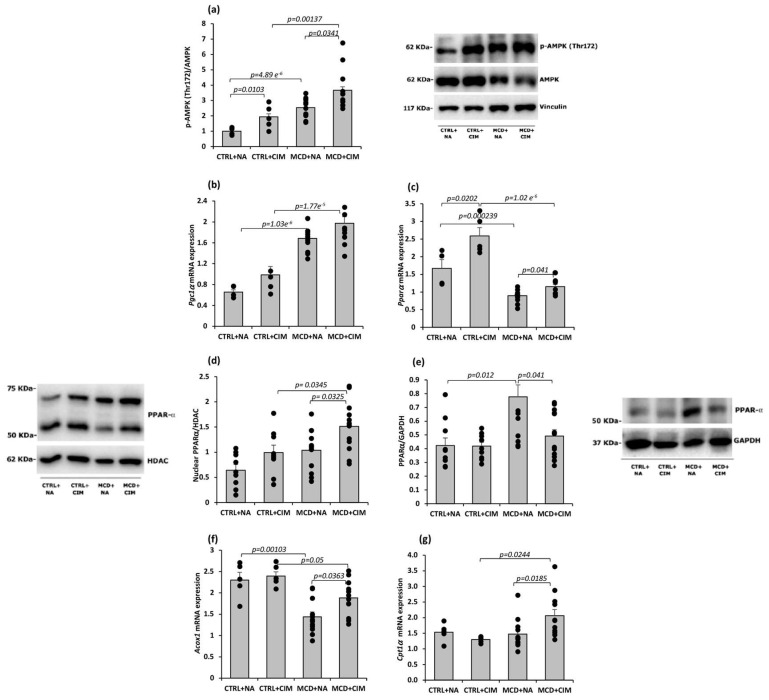
AMPK and PPARα protein expression was evaluated by Western blot. *Pgc1α*, *Pparα*, and its downstream genes’ (*Acox1* and *Cpt1α*) mRNA expression was assessed by RT-PCR in 6-week control or MCD-fed rats following CIM or vehicle administration. Rats were fed a control (CTRL) diet for 6 weeks and, starting from week 4, received daily oral administration of either CIM (10 mg/kg/day; CTRL + CIM, n = 6) or vehicle (CTRL + NA, n = 6). The same treatment regimen was applied to rats fed an MCD diet, resulting in two groups: MCD + CIM (n = 15) and MCD + NA (n = 15). (**a**) p-AMPK/AMPK ratio in hepatic tissue was significantly increased in both CIM-treated CTRL and MCD rats; (**b**) *Pgc1α* mRNA expression in hepatic tissue revealed a non-significant trend toward increased levels in CIM-treated groups; (**c**) *Pparα* mRNA expression in hepatic tissue increased significantly in both CIM-treated CTRL and MCD groups; (**d**) PPARα protein expression in hepatic nuclear fraction was marked increased in MCD rats administered with CIM; (**e**) PPARα protein expression in hepatic cytoplasmic fraction, consequently, significantly decreased in the same group; (**f**,**g**) *Acox1* and *Cpt1α* mRNA expression in hepatic tissue both increased in CIM-treated MCD rats. The results are expressed as mean ± S.E. of 15 biological replicates for MCD groups and 6 biological replicates for CTRL groups. Samples were loaded onto three separate polyacrylamide gels for Western blot analysis, while each sample was analyzed in triplicate both for target genes and housekeeping genes in RT-PCR analysis. CTRL = isocaloric control diet; MCD = methionine and choline-deficient diet; NA = Natrosol (vehicle); CIM = Complex I Modulator.

The effect of CIM on AMPK activation was first examined by Western blot analysis of the phosphorylated, active form of AMPK (p-AMPK Thr172). CIM treatment significantly increased p-AMPK levels in both control and MCD-fed rats compared with their respective vehicle-treated groups (Figure 4a).

AMPK promotes lipid catabolism, primarily by enhancing fatty acid oxidation via activation of PGC1α. PGC1α acts as a coactivator of PPARα, facilitating the transcription of genes involved in β-oxidation [61]. RT-PCR analysis revealed a non-significant trend toward increased *Pgc1α* mRNA levels in CIM-treated groups compared with vehicle-treated controls (Figure 4b). However, a significant increase in *Pparα* mRNA expression was observed in both control and MCD rats treated with CIM (Figure 4c), supporting the hypothesis that CIM promotes fatty acid oxidation via the AMPK–PGC1α–PPARα axis.

To further investigate this aspect, PPARα protein expression was evaluated in both the hepatic nuclear and cytoplasmic fractions. An increase in nuclear PPARα levels was observed in control rats treated with CIM, which became statistically significant in the MCD + CIM group compared with untreated MCD rats. Accordingly, PPARα levels in the cytoplasmic fraction were significantly reduced in CIM-treated MCD rats, further supporting this hypothesis (Figure 4d,e).

PPARα downstream genes, including peroxisomal acyl-coenzyme A oxidase 1 (*Acox1*) and the liver isoform of carnitine palmitoyltransferase 1α (*Cpt1α*), were also analyzed. The mRNA expression of both PPARα target genes was significantly increased in CIM-treated MCD rats, further confirming the role of CIM in enhancing β-oxidation (Figure 4f,g).

**Figure 5:**
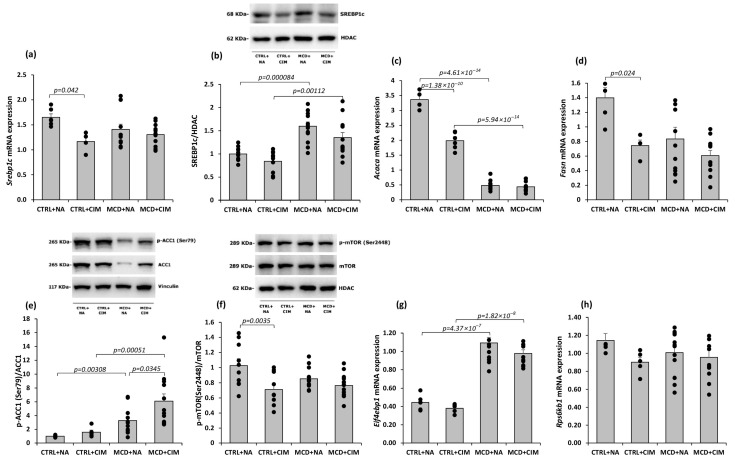
SREBP-1c mRNA and protein expression and its downstream gene (*Acaca* and *Fasn*) mRNA expression, p-ACC1 protein expression, mTORC1 nuclear protein expression, and *Eif4ebp1* and *Rps6kb1* mRNA expression in 6-week control or MCD-fed rats following CIM or vehicle administration. mRNA expression was assessed by RT-PCR analysis, while protein expression was measured by Western blot. Rats were fed a control (CTRL) diet for 6 weeks and, starting from week 4, received daily oral administration of either CIM (10 mg/kg/day; CTRL + CIM, n = 6) or vehicle (CTRL + NA, n = 6). The same treatment regimen was applied to rats fed an MCD diet, resulting in two groups: MCD + CIM (n = 15) and MCD + NA (n = 15). (**a**) *Srebp-1c* mRNA expression in hepatic tissue decreased significantly in CIM-treated CTRL rats; (**b**) SREBP-1c protein expression in hepatic nuclear fraction showed no significant changes after CIM administration; (**c**,**d**) *Acaca* and *Fasn* mRNA expression in hepatic tissue decreased significantly in CTRL rats treated with CIM; (**e**) p-ACC/ACC ratio in hepatic tissue was significantly increased in MCD rats after CIM administration; (**f**) p-mTOR/mTOR protein expression in hepatic nuclear fraction was significantly reduced in CTRL rats following CIM administration; (**g**,**h**) *Eif4ebp1* and *Rps6kb1* mRNA expression in hepatic tissue showed no significant changes after pharmacological treatment. The results are expressed as mean ± S.E. of 15 biological replicates for MCD groups and 6 biological replicates for CTRL groups. Samples were loaded onto three separate polyacrylamide gels for Western blot analysis, while each sample was analyzed in triplicate both for target genes and housekeeping genes in RT-PCR analysis. CTRL = isocaloric control diet; MCD = methionine and choline-deficient diet; NA = Natrosol (vehicle); CIM = Complex I Modulator.

To further verify the role of CIM in inhibiting the lipogenesis process via AMPK activation, both RNA and protein levels of the nuclear transcription factor SREBP-1c, as well as the mRNA levels of its target genes—acetyl-CoA carboxylase 1 (*Acaca*) and fatty acid synthase (*Fasn*)—were evaluated. As shown in Figure 5a, following CIM treatment *Srebp1c* mRNA expression was significantly reduced in the control group, while a non-significant reduction was observed in MCD rats. A similar trend was observed at the protein level for SREBP-1c in the hepatic nuclear fraction, although these changes did not reach statistical significance (Figure 5b). CIM was also associated with a significant decrease in mRNA expression of SREBP-1c target genes *Acaca* and *Fasn* in control rats. A similar but non-significant trend was observed in MCD rats (Figure 5c,d).

It is well known that AMPK directly inhibits lipogenesis by phosphorylating at Ser79 ACC1, a key enzyme in fatty acid synthesis. This process was also observed in our model: CIM significantly increased ACC phosphorylation at Ser79 in MCD rats, with the same trend observable also in control groups (Figure 5e).

The mechanistic target of rapamycin (mTOR) also contributes to the regulation of lipid metabolism; in fact, the activation of lipogenic SREBP-1c is largely mTOR-dependent [8,62]. Several studies have also demonstrated that AMPK and mTORC1 exert opposing effects on autophagy: while AMPK activates autophagy, mTORC1 suppresses it, contributing to lipid accumulation [61,63]. Phosphorylation of mTOR at Ser2448—a marker of mTORC1 activation—and the expression of its downstream targets, eukaryotic translation initiation factor 4E-binding protein 1 (4E-BP1) and ribosomal protein S6 kinase 1 (S6K1), were evaluated. CIM significantly reduced nuclear p-mTOR levels in control rats, with a similar but non-significant trend in MCD rats (Figure 5f). No significant changes were observed in the mRNA expression of 4E-BP1 and S6K1, although a slight reduction was noted in CIM-treated groups (Figure 5g,h).

### 3.5. CIM Reduces Hepatic Inflammation and Liver Fibrosis

**Figure 6:**
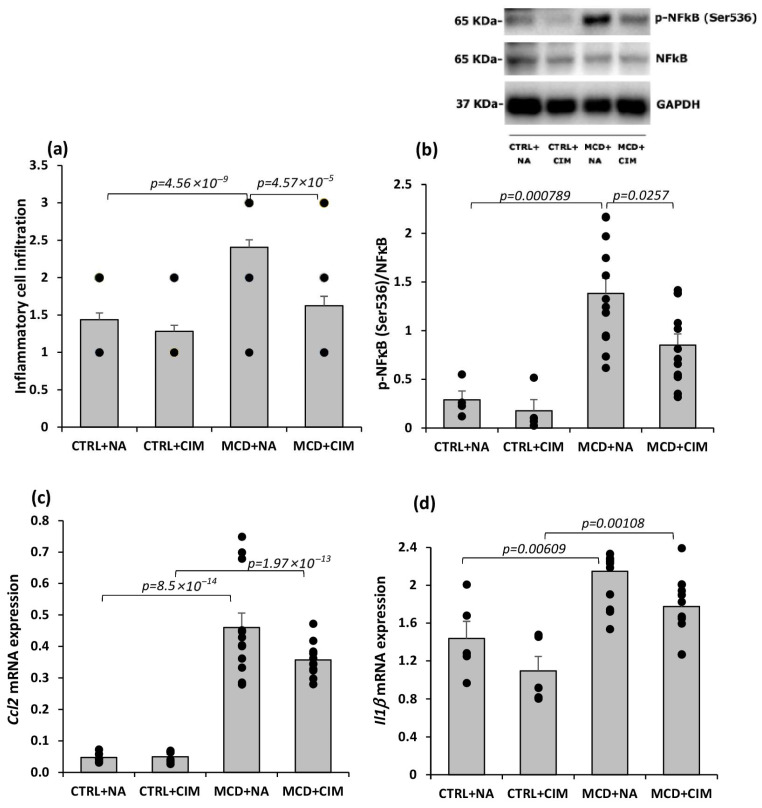
Inflammatory cell infiltration, p-NF-κB protein expression by Western blot analysis, and *Ccl2* and *Il1β* mRNA expression measured by RT-PCR in liver parenchyma of 6-week control or MCD-fed rats following CIM or vehicle administration. Rats were fed a control (CTRL) diet for 6 weeks and, starting from week 4, received daily oral administration of either CIM (10 mg/kg/day; CTRL + CIM, n = 6) or vehicle (CTRL + NA, n = 6). The same treatment regimen was applied to rats fed an MCD diet, resulting in two groups: MCD + CIM (n = 15) and MCD + NA (n = 15). (**a**) Inflammatory cell infiltration was assessed using a semi-quantitative histopathology score on liver sections stained by H&E. The graph showed a significant reduction in polymorphonuclear cell infiltration in MCD rats treated with CIM; (**b**) p-NF-κB/NF-κB ratio in whole hepatic tissue was significantly reduced in MCD rats after CIM administration; (**c**,**d**) *Ccl2* and *Il1β* mRNA expression in hepatic tissue decreased in CIM-treated MCD rats, although the reduction was not significant. The results are expressed as mean ± S.E. of 3 different fields of 4 different biological replicates for each group for inflammatory infiltration analysis. The results about western blot and RT-PCR are expressed as mean ± S.E. of 15 biological replicates for MCD groups and 6 biological replicates for CTRL groups. Samples were loaded onto three separate polyacrylamide gels for Western blot analysis, while each sample was analyzed in triplicate both for target genes and housekeeping genes in RT-PCR analysis. CTRL = isocaloric control diet; MCD = methionine and choline-deficient diet; NA = Natrosol (vehicle); CIM = Complex I Modulator.

Hematoxylin and eosin staining of liver sections revealed that the MCD diet led to a marked increase in inflammatory polymorphonuclear cell infiltration, whereas CIM administration significantly reduced this infiltration, restoring levels comparable to those observed in control rats (Figure 6a). Chronic activation of the nuclear factor kappa B (NF-κB) pathway is a hallmark of MASH and is linked to elevated circulating levels of pro-inflammatory cytokines such as tumor necrosis factor alpha (TNF-α), interleukin-6 (IL-6), and interleukin-1 beta (IL-1β) in obese individuals [64,65]. In our model, the MCD diet led to an increase in p-NF-κB (Ser536) levels, which were restored to control values following CIM administration, indicating an anti-inflammatory effect of CIM under these experimental conditions (Figure 6b). As expected, the MCD diet also induced an increase in the mRNA expression of the key inflammatory markers C-C motif chemokine ligand 2 (*Ccl2*) and interleukin-1 beta (*Il1β*). However, in CIM-treated rats, only a non-statistically significant trend toward reduced expression of these markers was observed (Figure 6c,d).

**Figure 7:**
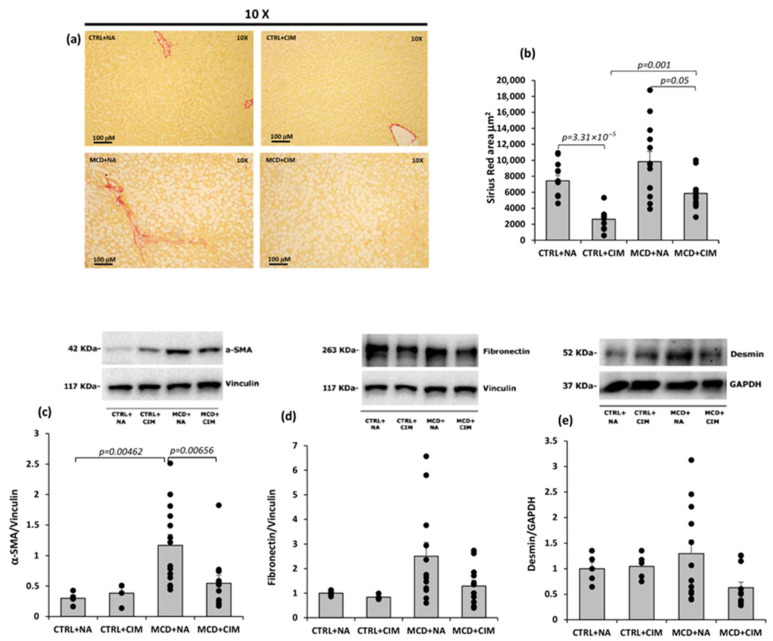
Evaluation of collagen fiber deposition by Sirius Red staining and α-SMA, fibronectin, and desmin protein expression evaluated in Western blot in livers from 6-week control or MCD-fed rats following CIM or vehicle administration. Rats were fed a control (CTRL) diet for 6 weeks and, starting from week 4, received daily oral administration of either CIM (10 mg/kg/day; CTRL + CIM, n = 6) or vehicle (CTRL + NA, n = 6). The same treatment regimen was applied to rats fed an MCD diet, resulting in two groups: MCD + CIM (n = 15) and MCD + NA (n = 15). (**a**) Sirius Red stained liver sections. Panels at 10X magnification are shown (scale bar: 100 μM). Slides were examined under a light microscope (Nikon Eclipse E800); (**b**) area of collagen fibers quantified with ImageJ software: a significant reduction was obtained in both CIM-treated animal groups; (**c**) α-SMA protein expression in whole liver extracts decreased significantly in MCD group after CIM administration; (**d**,**e**) fibronectin and desmin protein expression in whole liver extracts showed a similar trend toward reduction in MCD group treated with CIM. The results are expressed as mean ± S.E. of 3 different fields of 4 different biological replicates for each group for Sirius Red analysis, while the results are expressed as mean ± S.E. of 15 biological replicates for MCD groups and 6 biological replicates for CTRL groups for Western blot analysis. Samples were loaded onto three separate polyacrylamide gels for each target protein considered. CTRL = isocaloric control diet; MCD = methionine and choline-deficient diet; NA = Natrosol (vehicle); CIM = Complex I Modulator.

Sirius Red staining revealed that, in control groups, collagen deposition was largely restricted to blood vessels, with minimal staining in the parenchyma. In contrast, MCD-fed rats showed extensive collagen fiber accumulation throughout the liver tissue, which was markedly reduced by CIM treatment (Figure 7a), as confirmed by ImageJ-based quantification (Figure 7b).

The expression levels of alpha-smooth muscle actin (α-SMA), fibronectin, and desmin were assessed as markers of hepatic stellate cell (HSC) activation and fibrogenesis. In MCD-fed rats, hepatic α-SMA protein levels were markedly elevated, consistent with HSC activation and ongoing fibrotic remodeling. CIM treatment significantly reduced α-SMA expression, restoring it to levels comparable to those of control animals (Figure 7c). Fibronectin and desmin exhibited a similar trend, although these changes did not reach statistical significance (Figure 7d,e).

**Figure 8:**
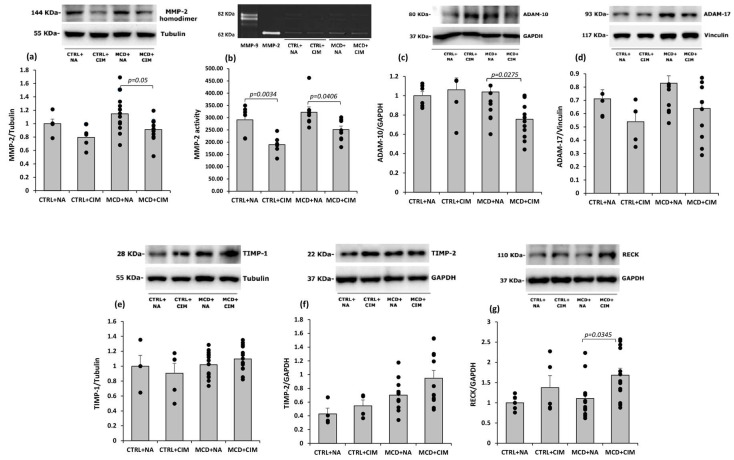
MMP-2, ADAM-10 and ADAM-17, TIMP-1, TIMP-2, and RECK protein expression was assessed by Western blot analysis, while MMP-2 activity was analyzed by zymography in the liver from 6-week control or MCD-fed rats following CIM or vehicle administration. Rats were fed a control (CTRL) diet for 6 weeks and, starting from week 4, received daily oral administration of either CIM (10 mg/kg/day; CTRL + CIM, n = 6) or vehicle (CTRL + NA, n = 6). The same treatment regimen was applied to rats fed an MCD diet, resulting in two groups: MCD + CIM (n = 15) and MCD + NA (n = 15). (**a**) MMP-2 protein expression in whole liver extracts showed a significant decrease in CIM-treated MCD rats; (**b**) MMP-2 activity assessed by zymography in whole liver extracts displayed a significant reduction in both CIM-treated CTRL and MCD groups; (**c**) ADAM-10 protein expression in whole liver extracts showed a significant reduction in MCD rats after pharmacological treatment with CIM; (**d**) ADAM-17 protein expression in whole liver extracts displayed a reduction, although not significant, in both CTRL and MCD rats treated with CIM; (**e**,**f**) TIMP-1 and TIMP-2 protein expression in whole liver extracts showed no significant changes among the considered experimental groups; (**g**) RECK protein expression in whole liver extracts showed a marked increase in MCD group after pharmacological treatment with CIM. The results are expressed as mean ± S.E. of 15 biological replicates for MCD groups and 6 biological replicates for CTRL groups. Samples were loaded onto three separate polyacrylamide gels for Western blot analysis and for gelatin zymography assay. CTRL = isocaloric control diet; MCD = methionine and choline-deficient diet; NA = Natrosol (vehicle); CIM = Complex I Modulator.

The hepatic extracellular matrix (ECM) is constantly remodeled during liver repair, but its dysregulation promotes fibrosis. Matrix metalloproteinases (MMPs), particularly MMP-2, are key mediators of ECM degradation [66]. While MCD feeding did not significantly alter MMP-2 levels, CIM treatment significantly reduced its protein expression (evaluated by Western blot) and enzymatic activity (assessed by zymography) (Figure 8a,b).

Several members of the disintegrin and metalloprotease (ADAM) family also play key roles in ECM remodeling, contributing to both pro- and anti-fibrotic processes. In particular, ADAM-10 and ADAM-17, released by activated HSCs, are involved in the shedding and release of chemotactic molecules, recruitment of inflammatory cells, and paracrine stimulation of HSCs themselves [44]. Our results show that CIM administration significantly reduced ADAM-10 protein expression in MCD rats (Figure 8c). Additionally, a trend for decrease in ADAM-17 protein expression was observed in both CIM-treated groups (*p* = 0.08) (Figure 8d). CIM administration did not affect tissue inhibitors of metalloproteinases (TIMP)-1 and -2 protein expression in both control and MCD rats (Figure 8e,f). On the contrary, the reversion-inducing cysteine-rich protein with Kazal motifs (RECK), which is a regulator of several MMPs as well as ADAM-10 and ADAM-17 [67], was significantly increased in CIM-treated MCD rats, with a similar trend also observed in treated control animals (Figure 8g).

Taken together, these data suggest that CIM may play a role in limiting fibrosis progression in our experimental model.

## 4. Discussion

Mitochondrial Complex I is a primary site of ROS generation, especially when there is an imbalance between substrate availability and ATP demand [22,23,24,25]. Its role in MASLD progression has gained further attention after discovering that metformin, a clinically used insulin sensitizer, exerts its beneficial effects through Complex I inhibition, leading to ROS and ATP decrease, and subsequent activation of AMPK, a key regulator of lipid metabolism [26,27]. Based on this rationale, our study investigated whether a novel selective complex I modulator (BI4500, CIM) could mitigate oxidative stress and lipid accumulation, improving inflammation and fibrosis. To this end, we administered CIM to both rats fed with an MCD diet and rats fed with a control diet supplemented with L-methionine and L-choline. In this study, we demonstrate that selective modulation of mitochondrial Complex I–derived reactive oxygen species by CIM markedly ameliorates key pathological features of MASH in MCD diet-fed rats. CIM treatment significantly reduced mitochondrial oxidative stress, improved mitochondrial efficiency, and attenuated hepatic inflammation and fibrotic remodeling. These effects were accompanied by a profound reprogramming of hepatic lipid metabolism, characterized by reduced steatosis, enhanced fatty acid oxidation, and suppression of lipogenic pathways, largely mediated by strong activation of AMPK signaling. Collectively, our findings identify selective Complex I modulation as an effective strategy to interrupt the vicious cycle linking mitochondrial dysfunction, oxidative stress, metabolic dysregulation, and fibrosis in experimental MASH.

### 4.1. Oxidative Stress and Redox Balance

In comparison with control rats, MCD-fed rats showed a dramatic increase in oxidative stress; CIM reduced oxidative stress in both CTRL and MCD rats. Interestingly, a CIM-mediated NOx reduction was evident in MCD and not in CTRL rats. This likely reflects the pathological upregulation of mtNOS in the MCD model [68]. In MCD livers, mtNOS activity is elevated and functionally coupled to complex I [69]; thus, RET complex I inhibition may be responsible for NO suppression. In contrast, control rats exhibit low mtNOS activity, and NOx levels are maintained by constitutive NOS isoforms, which are unaffected by mitochondrial modulation. These changes were accompanied by a marked increase in *Nqo1* expression in MCD rats in comparison with control rats, independently of CIM administration. NQO1 uses NAD(P)H to detoxify quinones, working as a compensatory mechanism to restore redox balance [70]. Signs of mitochondrial disruption were also evident in MCD rats, including a reduction in ATP and MIC-19—a key structural component of the MICOS complex [50,51,52]. Liver-specific knockout of MIC-19 in mice leads to severe disorganization of mitochondrial cristae and mitochondrial dysfunction, resulting in altered lipid metabolism and oxidative stress, which contributes to the exacerbation of MASH. Conversely, overexpression of MIC-19 in MCD mice reversed MASH [50]. Here, CIM administration significantly increased MIC-19 in MCD rats, suggesting that CIM may help to preserve mitochondrial architecture and function by stabilizing the MICOS complex. However, given the experimental design, it remains unclear whether MIC-19 restoration is a consequence of reduced oxidative stress or a contributing factor to it.

CIM, by reducing oxidative stress, induced a recovery of mitochondrial function, as evidenced by the improved RCR in CIM-treated MCD rats. Notably, studies in mouse models with genetic defects in mitochondrial OXPHOS have shown that reduced mitochondrial respiratory capacity can prevent MASH and improve insulin sensitivity [15]. Moreover, mice with complex I and V deficiencies, when fed with a high-fat diet, exhibit an increased RCR, as well as a reduced ATP content and lower ROS production, while their livers are protected from diet-induced steatosis and insulin resistance [71]. Interestingly, although CIM improved mitochondrial RCR, ATP levels were not significantly increased. This suggests that, in the context of advanced MCD-induced mitochondrial dysfunction, CIM primarily reduces mitochondrial ROS and restores redox homeostasis rather than directly boosting ATP production. Unlike simple antioxidants such as vitamin C, capable of scavenging free radicals [72,73], CIM targets the mitochondrial source of ROS while preserving respiratory function, which may explain the observed dissociation between ROS reduction and ATP levels.

Excess mitochondrial ROS places a high demand on antioxidant defenses, particularly the GSH system, which relies on NAD(P)H for GSH regeneration [74]. The oxidative burden in MCD rats increases NAD(P)H utilization, contributing to decreasing the NAD(P)H bound/free ratio [38]. A pharmacological intervention aimed at reducing mitochondrial ROS and the associated NAD(P)H demand may, in principle, restore the redox balance and influence the NAD(P)H bound/free ratio. The increased *Idh2* mRNA expression observed in CIM-treated MCD rats supports this mechanism, as IDH2 plays a key role in mitochondrial NAD(P)H production [53,54,55]. However, in our model, a reduction in the NAD(P)H bound/free ratio occurred only in CIM-treated control mice. In control animals, a relatively high NAD(P)H bound/free ratio indicates a metabolically active liver, requiring energy for anabolic and antioxidant processes. Upon CIM treatment, the ratio significantly decreased, likely reflecting a facilitated oxidation of NADH and a reduced demand for NAD(P)H-consuming detoxification enzymes due to lower mitochondrial ROS. Although a reduced NAD(P)H bound/free ratio is often seen as a sign of impaired mitochondrial function, its interpretation is context-dependent. In CIM-treated control animals, it is more likely to signal relief from oxidative pressure and an improved redox reserve, rather than dysfunction. In contrast, the markedly low NAD(P)H bound/free ratio observed in MCD rats reflects a severe redox collapse driven by overwhelming oxidative stress and impaired NAD(P)H regeneration [10]. Notably, CIM treatment in MCD-fed animals did not significantly alter the ratio, suggesting that, in this context of advanced mitochondrial dysfunction and persistent oxidative damage, the redox system remains unresponsive to CIM. This may reflect a threshold beyond which mitochondrial support alone is insufficient to restore redox homeostasis.

By maintaining mitochondrial redox homeostasis through NAD(P)H production, IDH2 indirectly affects lipid metabolism. In primary hepatocytes exposed to palmitate, reduced IDH2 expression led to marked lipid accumulation; on the contrary, overexpression of IDH2 reduced ROS production and restored the balance of mitochondrial fission and fusion mediators, ultimately leading to decreased dyslipidemia [53]. These findings were confirmed in IDH2 knockout mice fed a high-fat diet, which showed increased lipogenesis and reduced β-oxidation, changes likely driven by mitochondrial redox imbalance rather than by a direct modulation of β-oxidation [53].

In conclusion, our results thus far paint a clear picture of the MCD diet inducing a vicious cycle of mitochondrial dysfunction and oxidative stress, which CIM helps to interrupt. Key findings supporting this mechanism are that, in MCD rats, CIM administration significantly improved oxidative stress and mitochondrial integrity, and upregulated *Idh2* mRNA expression, compared with untreated counterparts. Given the role of IDH2 in maintaining mitochondrial redox balance, this finding supports the hypothesis that CIM enhances antioxidant defenses in our model. Beyond its effects on oxidative stress, modulation of mitochondrial Complex I may also influence broader metabolic pathways by altering mitochondrial redox balance and energy sensing. By limiting excessive Complex I–derived ROS production, CIM may indirectly affect NADH/NAD^+^ availability and downstream metabolic signaling, including AMPK activation. In this context, the metabolic changes observed in the present study—particularly those related to lipid handling—are consistent with a coordinated response linking redox homeostasis to metabolic regulation. Although a comprehensive metabolomic characterization of polar and non-polar metabolites was beyond the scope of this work, such approaches would be valuable in future studies to further delineate the systemic metabolic consequences of selective Complex I modulation. Notably, since IDH2 activity is associated with improvements in hepatic lipid metabolism, we next aimed to investigate whether its upregulation might also contribute to the control of lipid accumulation in MCD rats.

### 4.2. Lipid Accumulation and AMPK Pathway

To validate this hypothesis, we assessed lipid content in our MASLD model. Firstly, we observed a marked increase in lipid accumulation and macrosteatosis in MCD rats, consistent with previous literature [75,76]. We also found that CIM significantly reduced hepatic fat content and lipid droplet area, with the most pronounced effect observed in the steatotic condition, suggesting a therapeutic potential in reversing diet-induced steatosis. The central node of this action appears to be a profound reprogramming of lipid metabolism mediated by the potent activation of AMPK, the cell’s master metabolic sensor and key regulator of lipid metabolism [77,78,79]. Notably, in the MCD group, which already exhibited elevated p-AMPK due to cellular stress, CIM induced a convergent and potentially additive increase, highlighting its robust and direct effect on this pathway. This potent AMPK activation triggered a coordinated, two-pronged metabolic shift. Firstly, CIM stimulated fatty acid catabolism: while the MCD diet suppressed the key transcriptional regulator of β-oxidation, PPARα, CIM acted antagonistically to the diet, effectively reversing this suppression by significantly increasing both *Pparα* mRNA expression and its nuclear translocation. This functional activation was confirmed by the corresponding upregulation of its downstream target genes, including *Acox1* and *Cpt1α*, thereby reactivating the machinery for fatty acid oxidation. Secondly, and in stark contrast to its effect on β-oxidation, CIM strongly inhibited anabolic pathways, a mechanism that involved mTOR inhibition and the downregulation of the mTOR downstream signaling cascade as well.

Given its central role, AMPK is considered a key factor in the pathophysiology of MASLD. AMPK activation counteracts MASLD progression by inhibiting hepatic lipogenesis, enhancing fatty acid oxidation in the liver, and improving mitochondrial function in the liver and adipose tissue [79,80]. Both genetic and pharmacological approaches aimed at activating AMPK have demonstrated beneficial effects in experimental models of MASLD [81,82]. Notably, metformin—an inhibitor of Complex I—exerts its beneficial effects primarily via AMPK activation [83]. Active AMPK enhances fatty acid oxidation by upregulating PGC1α, PPARα, and CPT1, while simultaneously inhibiting lipogenesis through downregulation of ACC1, FAS, and SREBP-1c [61]. Data from literature also show that metformin inhibits mTORC1 via AMPK, consequently decreasing SREBP-1c—a key transcription factor driving lipid accumulation [62,84]. Our data support this notion, showing a significant reduction in p-mTOR in CIM-treated control animals. However, in the MCD group, mTOR signaling was already suppressed, likely as a consequence of the severe metabolic stress. This created a “floor effect,” where the already low baseline masked the additional inhibitory contribution of CIM, preventing the observation of a further statistically significant reduction. A similar floor effect was evident at the transcriptional level of lipogenic genes: the MCD diet itself markedly suppressed the expression of *Srebp-1c*, *Acaca*, and *Fasn*; while CIM clearly repressed these genes in healthy controls, its effect was not statistically apparent in the MCD cohort, as their expression had already been maximally suppressed by the diet. Despite this transcriptional floor effect, the powerful functional blockade of de novo lipogenesis by CIM was unequivocally demonstrated by the massive increase in the phosphorylation of ACC1 (p-ACC1), which renders the key lipogenic enzyme inactive. Considering that in MCD animals the CIM-mediated effect on mTOR and SREBP is not significant, we suggest that the increase in phosphorylated (inactive) ACC1 was directly mediated by AMPK, as already described in the literature [85].

### 4.3. Inflammation and Fibrosis

Hepatic inflammation is a hallmark of MASH, and the MCD diet is a well-established model to trigger pronounced inflammation and fibrosis in the liver [86,87]. In particular, several lines of evidence have reported that the MCD diet leads to the upregulation of the proinflammatory mediator NF-κB [88]. Sustained activation of NF-κB, the key factor in the control of inflammation [89,90], is a defining feature of MASH and has been associated with increased circulating levels of pro-inflammatory cytokines in obese individuals [69,71]. Hepatocyte-specific knockdown of NF-κB has been shown to protect against diet-induced steatosis in mice [74]. A trigger of NF-κB activation is lipotoxicity, which can promote inflammation through several mechanisms. First, an excess of free fatty acids in hepatic tissue promotes lysosomal permeabilization and cathepsin B release, which in turn activates NF-κB. Indeed, cathepsin B levels in MASH patients correlate with a high degree of inflammation, whereas its deficiency protects mice from diet-induced fatty liver despite the presence of obesity [91,92]. Second, lipotoxicity induces ER stress, which also activates NF-κB [92]. Finally, fatty acids can activate Toll-like receptor 4 (TLR4), leading to NF-κB activation and upregulation of its target genes, TNF-α and IL-6. In MCD-fed mice, TLR4 activation results in increased levels of TNF-α, IL-6, and IL-12, while TLR4 deficiency reduces liver injury by decreasing triacylglycerol accumulation and inflammatory gene expression [92]. Our data are in line with these mechanisms and showed a significant reduction in NF-κB activation in CIM-treated MCD rats, accompanied by decreased, although not significantly, expression of key inflammatory markers such as *Ccl2* and *Il1β*. These findings suggest that CIM reduces inflammation in our MASH model by acting on both redox balance and lipotoxicity.

Inflammation and lipotoxicity are also key driving mechanisms in the development of liver fibrosis. NF-κB activation is considered a critical link between inflammation, fibrosis, and hepatocellular carcinoma (HCC) [93,94]. When excessive ROS levels and, consequently, DAMPs are present, the latter activate TLR4, ultimately leading to NF-κB activation; the released proinflammatory cytokines activate HSCs, triggering fibrogenesis [94,95,96,97]. Lipid accumulation, on the other hand, promotes lipotoxicity and AMPK inactivation, as observed also in our model. Notably, AMPK not only regulates lipid metabolism but also plays a crucial role in inactivating HSCs. AMPK-mediated autophagy, via the AMPK/mTOR pathway, alleviates liver fibrosis by promoting HSC apoptosis [94,98]. In our model, CIM administration led to a reduction in NF-κB activation, accompanied by strong AMPK activation. This dual action contributed to decreasing inflammation and lipid accumulation, ultimately inhibiting fibrosis development. Supporting CIM’s antifibrotic role, we observed reduced collagen deposition in hepatic parenchyma, associated with decreased expression of key fibrotic and HSC activation markers, including α-SMA, fibronectin, and desmin—major components of the extracellular matrix (ECM).

As a consequence of the CIM-induced reduction in fibrosis development, molecular pathways involved in collagen deposition were modulated in our model. Matrix metalloproteinases (MMPs) and their specific tissue inhibitors (TIMPs) play a crucial role in ECM remodeling, and a disruption in the balance between MMP and TIMP activity exacerbates fibrotic damage [66]. Numerous studies have demonstrated that increased MMP-2 activity is closely associated with fibrosis progression [44,66,67,99]. In our model, MMP-2 protein expression and activity, elevated in MCD rats, were restored to control levels following CIM administration, along with the sheddases ADAM-10 and ADAM-17. These members of the ADAM family play key roles in ECM remodeling [100]. The expression of ADAM-10 and ADAM-17 increases in parallel with HSC activation [101]. ADAM-10 promotes the shedding and release of pro-inflammatory molecules such as Notch [102], and activates HSCs [103]. ADAM-17, on the other hand, is directly involved in the release of TNF-α, which can contribute to the progression of MASH [67]. Indeed, it has been shown that downregulation of ADAM-17—whether genetically or pharmacologically induced—protects high-fat diet (HFD)-fed mice from obesity, insulin resistance, and diabetes [104,105]. Finally, ADAM-10, ADAM-17, and MMP-2 released by activated HSCs promote the shedding and release of CX3CL1, enabling inflammatory cell recruitment and HSC activation, thereby contributing to chronic liver disease progression [106]. In line with these findings, we observed a trend toward reduced ADAM-17 protein expression in CIM-treated MCD rats (*p* = 0.08).

Also, TIMP-1 and TIMP-2 expression is strongly linked to fibrosis progression [107,108]. However, in our study, we did not observe significant changes in TIMP-1/2 expression among the experimental groups, regardless of pharmacological treatment. This lack of effect may be due to the degree of fibrosis reached in our model: animals were challenged with either an MCD or an isocaloric control diet for 6 weeks, a duration that is not sufficient to induce advanced liver fibrosis. Numerous preclinical and clinical studies confirm that TIMP-1 expression increases progressively during liver fibrosis development, peaking in the cirrhotic stage [109,110].

MMPs and ADAMs are also inhibited by RECK [67], a negative regulator whose expression is reduced in MASH models [111,112,113], exacerbating steatosis, fibrosis, and inflammation. RECK downregulation was associated with increased expression of fibrogenic genes such as TGF-β, collagen I, MMPs, TIMP-1, and ADAM10/17 [44].

In our study, CIM administration upregulated RECK protein levels, further supporting its protective role against liver inflammation and fibrosis.

## 5. Conclusions, Limitations of the Study, and Translational Implications

In conclusion, our data collectively demonstrate that selective modulation of complex I represents a promising strategy to counteract the progression of MASLD toward MASH. In our model, administration of CIM helps to disrupt the vicious cycle of mitochondrial dysfunction and oxidative stress, thereby improving mitochondrial integrity and function. This improved pathological environment contributes to reducing hepatic fat accumulation by reprogramming lipid metabolism via the AMPK pathway, ultimately attenuating inflammation and key fibrogenic drivers.

Despite these findings, the present study has some limitations. Although the MCD diet model is suitable for investigating steatohepatitis and fibrosis, it does not fully recapitulate the metabolic context of human MASLD, particularly with respect to obesity, insulin resistance, and systemic metabolic dysfunction. Moreover, the use of a single terminal time point precluded assessment of disease progression and long-term treatment effects. In addition, the absence of individual baseline measurements did not allow the evaluation of intra-subject changes over time, which would have provided further insight into treatment-induced variations. While our data support a role for AMPK activation, causality cannot be definitively established without targeted mechanistic interventions, and future studies employing genetic or pharmacological AMPK inhibition will be required to confirm this mechanism. In addition, the translational relevance of selectively modulating mitochondrial Complex I–derived ROS will need to be validated in additional preclinical models that more closely mimic human MASLD and in longer-term studies assessing safety and efficacy.

Regardless of these limitations, the present findings provide important translational insights. By selectively suppressing pathological mitochondrial ROS production without compromising mitochondrial respiration, BI4500 represents a mechanistically targeted approach that may overcome limitations associated with non-specific antioxidants or classical mitochondrial inhibitors. The ability of BI4500 to simultaneously improve mitochondrial function, reprogram hepatic lipid metabolism, and attenuate inflammation and fibrosis highlights selective Complex I modulation as a viable therapeutic strategy. Future studies in metabolically relevant preclinical models and long-term intervention settings will be essential to define the translational potential of Complex I modulators for the management of MASLD and the prevention of disease progression.

## Figures and Tables

**Table 1:** Basic phenotyping information about body weight and liver weight. 6-week CTRL or MCD-fed rats following CIM or vehicle administration were used. Data for MCD groups are presented as mean ± S.E. from 15 biological replicates; CTRL groups represent mean ± SEM from 6 biological replicates. CTRL = isocaloric control diet; MCD = methionine and choline-deficient diet; NA = Natrosol (vehicle); CIM = Complex I Modulator.

Phenotyping Information	CTRL + NA	CTRL + CIM	MCD + NA	MCD + CIM
Body weight	463.3 ± 14.4	468.8 ± 9.2	210.6 ± 3.6 ^b^***	211.3 ± 2.5 ^a^***
Liver weight	16.1 ± 0.8	17.9 ± 0.6	10.8 ± 0.3 ^b^***	11.2 ± 0.25 ^a^***

^a^ CTRL + CIM versus MCD + CIM; ^b^ CTRL + NA versus MCD + NA. The level of significance is indicated by asterisks: *** *p* ≤ 0.001.

**Table 2:** Hepatic enzyme release and model characterization. Six-week CTRL or MCD-fed rats following CIM or vehicle administration were used. AST and ALT were measured using a UV kinetic method, while ALP was assessed using a colorimetric kinetic method. Total bilirubin was determined by a colorimetric assay, and cholesterol and triglycerides were quantified using a colorimetric enzymatic method. All these assays were performed with a Beckman Coulter AU 5820 instrument. LDH enzymatic activity was measured spectrophotometrically at 340 nm in the presence of saturating concentrations of NADH and pyruvate, using a T92^+^ UV spectrophotometer. Data for MCD groups are presented as mean ± S.E. from 15 biological replicates; CTRL groups represent mean ± SEM from 6 biological replicates. CTRL = isocaloric control diet; MCD = methionine and choline-deficient diet; NA = Natrosol (vehicle); CIM = Complex I Modulator.

Plasma Parameters	CTRL + NA	CTRL + CIM	MCD + NA	MCD + CIM
AST (IU/L)	79.8 ± 14.9	43.6 ± 1.5 ^a^*	130 ± 11.1 ^c^**	157.2 ± 10.7 ^b^***
ALT (IU/L)	40 ± 8.7	21.4 ± 0.7 ^a^*	173.6 ± 9.2 ^c^***	195.3 ± 16.5 ^b^***
ALP (IU/L)	17.6 ± 4.1	8.2 ± 1.9	41 ± 5.7	45.2 ± 10.3 ^b^***
LDH (mU/mL)	4.4 ± 1.3	0.94 ± 0.17 ^a^**	5.4 ± 1.0	4.9 ± 0.68 ^b^***
Total Bilirubin (mg/dL)	0.10 ± 0.02	0.098 ± 0.02	0.46 ± 0.07 ^c^***	0.51 ± 0.09 ^b^**
Cholesterol (mg/dL)	52.5 ± 3.8	51.8 ± 3.1	18.1 ± 2.1 ^c^***	18.4 ± 1.2 ^b^**
Triglycerides (mg/dL)	229.8 ± 33.7	129 ± 22.6 ^a^**	9.9 ± 0.6 ^c^***	12.3 ± 0.9 ^b^***

^a^ CTRL + NA versus CTRL + CIM; ^b^ CTRL + CIM versus MCD + CIM; ^c^ CTRL + NA versus MCD + NA. Different levels of significance are indicated by asterisks: * *p* ≤ 0.05; ** *p* ≤ 0.01; *** *p* ≤ 0.001.

## Data Availability

The original contributions presented in this study are included in the article. Further inquiries can be directed to the corresponding authors.

## Abbreviations

The following abbreviations are used in this manuscript:

ACC1	Acetyl-CoA Carboxylase 1
ACC2	Acetyl-CoA Carboxylase 2
ACOX1	Acyl-CoA oxidase 1
ADAM-10	Disintegrin and Metalloproteinase Domain-Containing Protein 10
ADAM-17	Disintegrin and Metalloproteinase Domain-Containing Protein 17
ADP	Adenosine Diphosphate
ALP	Alkaline Phosphatase
ALT	Alanine Aminotransferase
AMP	Adenosine Monophosphate
AMPK	AMP-activated Protein Kinase
AST	Aspartate Transaminase
ATP	Adenosine Triphosphate
BSA	Bovine Serum Albumin
CCL2	C-C Motif Chemokine Ligand 2
CIM	Complex I Modulator
CPT1	Carnitine Palmitoyltransferase 1
CTRL	Control
DCFH	Dihydro-Dichlorofluorescein
DCFH-DA	Dihydro-Dichlorofluorescein Diacetate
DMN	Dimethylnitrosamine
4E-BP1	Eukaryotic Translation Initiation Factor 4E-binding Protein 1
ECM	Extracellular Matrix
ER	Endoplasmic Reticulum
FADH_2_	Flavin Adenine Dinucleotide, Reduced
FAS	Fatty Acid Synthase
FZHY	Fuzhenghuayu
H&E	Hematoxylin and Eosin
HCC	Hepatocellular carcinoma
HO-1	Heme Oxygenase-1
HSCs	Hepatic Stellate Cells
IDH2	Isocitrate Dehydrogenase 2
IL-1β	Interleukin 1 beta
IL-6	Interleukin 6
LDH	Lactate Dehydrogenase
MAFLD	Metabolic Dysfunction-Associated Fatty Liver Disease
MASLD	Metabolic Dysfunction-Associated Steatotic Liver Disease
MCD	Methionine and Choline Deficient Diet
MDA	Malondialdehyde
MICOS	Mitochondrial Contact Site and Cristae Organizing System
MMP-2	Metalloproteinase 2
MMPs	Metalloproteinases
mtDNA	Mitochondrial DNA
mtNOS	Mitochondrial Nitric oxide synthase
mTOR	Mechanistic Target of Rapamycin
mTORC1	Mechanistic Target of Rapamycin Complex 1
NA	Natrosol
NAD(P)H	Nicotinamide Adenine Dinucleotide Phosphate, reduced form
NADH	Nicotinamide Adenine Dinucleotide
NAFLD	Directory of open access journals
NF-κB	Nuclear Factor kappa B
NQO1	NAD(P)H Dehydrogenase Quinone 1
NRF2	Nuclear Transcription Factor Erythroid 2–Related Factor 2
OXPHOS	Oxidative Phosphorylation
p-NF-κB	Nuclear Factor kappa B, phosphorylated
p-ACC1	Acetyl-CoA Carboxylase 1, phosphorylated
p-AMPK	AMP-activated Protein Kinase, phosphorylated
PGC1α	Peroxisome Proliferator-Activated Receptor Gamma Coactivator 1-alpha
PPAR-α	Peroxisome Proliferator-Activated Receptor alpha
RCR	Respiratory Control Ratio
RECK	Reversion-inducing Cysteine-rich Protein with Kazal motifs
RET	Reverse Electron Transport
ROS	Reactive Oxygen Species
α-SMA	Alpha-Smooth Muscle Actin
S6K1	Ribosomal Protein S6 Kinase 1
SREBP-1c	Sterol Regulatory Element Binding Protein-1c
TACE	TNF-α–Converting Enzyme
TBARS	Thiobarbituric Acid Reactive Substance
TBS-T	Tris-Buffered Saline with Tween20
TCA	Tricarboxylic Acid Cycle
TGF-β	Transforming Growth Factor-β
THR-β	Thyroid Hormone Receptor β
TIMP-1	Tissue Inhibitor of Metalloproteinase, 1
TIMP-2	Tissue Inhibitor of Metalloproteinase, 2
TIMPs	Tissue Inhibitor of Metalloproteinases
TLR4	Toll-like receptor 4
TNF-α	Tumor Necrosis Factor alpha
UCP2	Uncoupling Protein 2
VLDL	Very Low Density Lipoprotein

## Appendix A

### Appendix A.1. MCD Diet and Isocaloric Control Diet Compositions

The composition of the Methionine and Choline Deficient (MCD) diet and the isocaloric control diet is expressed in mg/kg of diet. The control diet is identical to the MCD diet in terms of caloric content, but it is supplemented with L-methionine and L-choline (Table A1).

**Table A1:** Methionine and choline-deficient diet and isocaloric control diet compositions.

Ingredients	MCD (mg/kg)	Isocaloric Control Diet (mg/kg)
Protected Vitamin A 1000	40	40
Vitamin D3 500	5	5
Protected Vitamin E	121	121
L-Alanine	3500	3500
L-Arginine Hydrochloride	12,100	12,100
L-Asparagine Monohydrate	6000	6000
L-Aspartic Acid	3500	3500
L-Cystine	3500	3500
L-Glutamic Acid	40,000	40,000
Glycine	23,300	23,300
L-Histidine Hydrochloride	4500	4500
L-Isoleucine	8200	8200
L-Leucine	11,100	11,100
L-Lysine Hydrochloride	18,000	18,000
L-Phenylalanine	7500	7500
L-Proline	3500	3500
L-Serine	3500	3500
L-Threonine	8200	8200
L-Tryptophan	1800	1800
L-Tyrosine	5000	5000
L-Valine	8200	8200
L-Choline	-	2000
L-Methionine	-	3000
Sucrose	455,414	455,414
Dicalcium phosphate	3000	3000
Vitamin mix	5020	5020
Mineral mix AIN-76	35,000	35,000
Corn Starch	200,000	200,000
Corn Oil	100,000	100,000
Cellulose (Aphacel)	30,000	30,000

### Appendix A.2. Primary and Secondary Antibodies Used in This Study

Primary and secondary antibodies with appropriate dilutions and suppliers applied in this study for Western blot analysis are listed in Table A2.

**Table A2:** Primary and secondary antibodies, suppliers, and dilutions.

	Primary Antibody	Secondary Antibody
Mouse monoclonal antibodies	ADAM-10 (Santa Cruz Biotechnology, Inc.) 1:200	1:1000
	Desmin (Santa Cruz Biotechnology, Inc.) 1:5000	1:2000
	HDAC (Cell Signaling Technology) 1:1000	1:2000
	MMP-2 (Thermo Scientific) 1:200	1:1000
	NFκB (Santa Cruz Biotechnology, Inc.) 1:1000	1:1000
	PPARα (Santa Cruz Biotechnology, Inc.) 1:1000	1:1000
	SREBP-1c (Santa Cruz Biotechnology, Inc.) 1:500	1:1000
	TIMP-1 (Santa Cruz Biotechnology, Inc.) 1:500	1:1000
	Tubulin (Merck Millipore) 1:1000	1:2000
	Vinculin (Santa Cruz Biotechnology, Inc.) 1:1000	1:2000
Rabbit monoclonal antibodies	p-ACC (Cell Signaling Technology) 1:1000	1:2000
	ACC (Cell Signaling Technology) 1:1000	1:2000
	p-AMPK (Cell Signaling Technology) 1:1000	1:1000
	AMPK (Cell Signaling Technology) 1:1000	1:1000
	p-mTOR (Cell Signaling Technology) 1:1000	1:1000
	mTOR (Cell Signaling Technology) 1:1000	1:1000
	p-NFκB (Abcam) 1:1000	1:2000
	RECK (Cell Signaling Technology) 1:1000	1:1000
	α-SMA (Cell Signaling Technology) 1:1000	1:2000
Rabbit polyclonal antibodies	ADAM-17 (GeneTex) 1:1000	1:1000
	Fibronectin (Abcam) 1:1000	1:1000
	GAPDH (Proteintech) 1:20000	1:6000
	MIC-19 (Abcam) 1:1000	1:2000
	TIMP-2 (Abcam) 1:1000	1:2000

### Appendix A.3. Primers, Unique Assay ID, and Amplicon Sequences Used in RT-PCR Analysis

The amplicon sequences with additional base pairs added to the beginning and/or end of the sequence, in accordance with the minimum information for the publication of real-time quantitative PCR experiments (MIQE) guidelines, as reported by Bustin et al., are listed in Table A3.

**Table A3:** Amplicon sequences of primers used in this study. Acetyl-Coenzyme A carboxylase alpha (*Acaca*); acyl-Coenzyme A oxidase 1, palmitoyl (*Acox1*); C-C motif chemokine ligand 2 (*Ccl2*); carnitine palmitoyltransferase 1 alpha, (liver isoform, *Cpt1a*); eukaryotic translation initiation factor 4E binding protein 1 (*Eif4ebp1*); fatty acid synthase (*Fasn*); isocitrate dehydrogenase 2 (*Idh2*); interleukin 1 beta (*Il1β*); NAD(P)H dehydrogenase quinone 1 (*Nqo1*); nuclear factor (erythroid-derived 2)-like 2 (*Nfe2l2* or *Nrf2*); peroxisome proliferator-activated receptor gamma coactivator 1-alpha (*Ppargc1a*); peroxisome proliferator receptor alpha (*Pparα*); ribosomal protein S6 kinase, polypeptide 1 (*Rps6kb1*); sterol regulatory element binding transcription factor 1 (*Srebf1* or *Srebp-1c*) and mitochondrial uncoupling protein 2 (*Ucp2*) were the genes of interest to be analyzed. Glyceraldehyde-3-phosphate dehydrogenase (*Gapdh*) and hypoxanthine phosphoribosyltransferase 1 (*Hprt1*) were used as reference genes.

Gene	Amplicon Sequence
*Acaca* (ID: qRnoCID0001546)	TCAGCCTGTATAAGGAAGTGACTGACTCCAGGACAGCACAGATCATGTTTCAGGCGTATGGAGACAAGCAGGGACCACTGCATGGAATGTTAATCAATACTCCGTATG TGACCAAAGACCTTCTTCAATCAAAGAGGTTTCAG
*Acox1* (ID: qRnoCID0005662)	AATGGATGCGCCCGTCCCAAGAACTCCAGATAATTGGCACCTACGCCCAGACGGAGATGGGCCACGGAACTCATCTTCGAGGCTTGGAAACCACTGCCACATATGACC CCAAGAC
*Ccl2* (ID: qRnoCED0009272)	TAAATCTGAAGCTAATGCATCCACTCTCTTTTCCACAACCACCTCAAGCACTTCTGTAGAAGTGACCAGTATGACAGAGAACTAGTGTGATTTGGAATGTGATGCCTTAAG TAATGTTAAACTTATTTAACT
*Cpt1a* (ID: qRnoCID0009604)	TGCCTCTATGTGGTGTCCAAGTATCTTGCAGTCGACTCACCTTTCCTGAAGGAGGTATTGTCTGAGCCATGGAGGTTGTCTACGAGCCAGACTCCTCAGCAGCAGGTGG AGCTCTTTGACTT
*Eif4ebp1* (ID: qRnoCID0004845)	CTGCGTCCTATGGCTGGTCCCTTAAATGTCCATCTCAAACTGTGACTCTTCACCACCTGCCCGCTTATCTTCCGGGCTGCTGTGCAGATGGCTCTGGCTGGCCTGCATG GGAGGCTCATCGCTGGTAGGGCTAGTGACCCCTGGAATGGT
*Fasn* (ID: qRnoCED0004370)	TGCTCTCTGTGGATAGGACTGAATGCTGTGGCCTTCTGATAGACTCTTCTGGACA CTCAAGCCTGGTCAGTTTTGTGTGACTCAGGTAGGCA
*Gapdh* (ID: qRnoCID0057018)	TGATGGCAACAATGTCCACTTTGTCACAAGAGAAGGCAGCCCTGGTAACCAGGCGTCCGATACGGCCAAATCCGTTCACACCGACCTTCACCATCTTGTCTATGAGACGAGGCTGGCACTGCACA AGAAGATGCGGCTGTCTCTA
*Hprt1* (ID: qRnoCED0057020)	TTCATGCAAAAGCTTTACTAAGTAGATGGCCACAGGACTAGAACGTCTGCTAGTTCTTTACTGGCCACATCAACAGGACTCTTGTAGATTCAACTTGCCGCTGTCTTTT
*Idh2* (ID: qRnoCED0006136)	CCTGGAAGATGTCCTTGAAACGCCCGTCATAGGCCTTCATAATGGTGTTCTTGGT GCTCAAGTAGAGCGGCCATTTCTTCTGGATAGAGTACTGGAAGCAGCT
*Il1β* (ID: qRnoCID0004680)	TGAAAGCTCTCCACCTCAATGGACAGAACATAAGCCAACAAGTGGTATTCTCCATGAGCTTTGTACAAGGAGAGACAAGCAACGACAAAATCCCTGTGGCCTTGGGCCT CAAGGGGAAGAATCTATACCTGTCCTGTGTGATGAAAGACG
*Nqo1* (ID: qRnoCID0001006)	CAATCAGCGCTTGACACTACGATCCGCCCCCAACTTCTGGAGCCATGGCGGTGAGAAGAGCCCTGATTGTATTGGCCCACGCAGAGAGGACATCATTCAACTATGCCA TGAAGGAGGCTGCTGTGGAGGCTCTGAAGAAGAAAGGA
*Nfe2l2 (Nrf2)* (ID: qRnoCID0008218)	TACCCCAAGATCTATGTCTTGCCTCCAAAGGATGTCAATCAAATCCATGTCCTGCTGGGACTGTAGTCCTGGCGGTGGCAATTCCAAGTCCATCATGCTGAGGGCGGACGCTGCGCTAGGGC
*PPARgc1a (Pgc1a)* (ID: qRnoCID0001024)	TCATATCCAACCAGTACAACAATGAGCCCGCGAACATATTCGAGAAGATAGATGAAGAGAATGAGGCAAACTTGCTAGCGGTTCTCACAGAGACACTGGACAGTCTCCC GGTGGATGAAGACGGATTGCCCT
*Ppara* (ID: qRnoCID0004661)	CAAGGCCTCAGGATACCACTATGGAGTCCACGCATGTGAAGGCTGCAAGGGCTTCTTTCGGCGAACTATTCGGCTAAAGCTGGCGTACGACAAGTGTGATCGAAGCTG CAAGATTCAGAAAAAGAACCGGAACAAATGCCAGT
*Rps6kb1* (ID: qRnoCID0002512)	GGCATTTACATCAAAAAGGGATCATCTACAGAGACCTGAAGCCGGAGAACATCATGCTTAATCACCAAGGTCACGTGAAGCTGACAGACTTTGGACTATGCAAAGAATC TATTCATGATGGAACAGTCACGCACACATTTTGTGGAACAA
*Srebf1 (Srebp-1c)* (ID: qRnoCID0003286)	GTCACTGTCTTGGTTGTTGATGAGCTGAAGCATGTCTTCGATGTCGGTCAAGAG CGCCGCGTCCATTTCGCACACCTCGGCCAGTGCCTGTTCCAGAGC
*Ucp2* (ID: qRnoCED0009215)	TCATGACAGACGACCTCCCTTGCCACTTCACTTCTGCCTTCGGGGCGGGCTTCTGCACCACCGTCATTGCCTCCCCCGTTGATGTGGTCAAGACGAGATATATGAACT CTGC

## References

[1] Abdelhameed F., Kite C., Lagojda L., Dallaway A., Chatha K.K., Chaggar S.S., Dalamaga M., Kassi E., Kyrou I., Randeva H.S. (2024). Non-Invasive Scores and Serum Biomarkers for Fatty Liver in the Era of Metabolic Dysfunction-Associated Steatotic Liver Disease (MASLD): A Comprehensive Review From NAFLD to MAFLD and MASLD. Curr. Obes. Rep..

[2] Chan W.-K., Chuah K.-H., Rajaram R.B., Lim L.-L., Ratnasingam J., Vethakkan S.R. (2023). Metabolic Dysfunction-Associated Steatotic Liver Disease (MASLD): A State-of-the-Art Review. J. Obes. Metab. Syndr..

[3] Younossi Z.M., Koenig A.B., Abdelatif D., Fazel Y., Henry L., Wymer M. (2016). Global Epidemiology of Nonalcoholic Fatty Liver Disease-Meta-Analytic Assessment of Prevalence, Incidence, and Outcomes. Hepatology.

[4] Riazi K., Azhari H., Charette J.H., Underwood F.E., King J.A., Afshar E.E., Swain M.G., Congly S.E., Kaplan G.G., Shaheen A.-A. (2022). The Prevalence and Incidence of NAFLD Worldwide: A Systematic Review and Meta-Analysis. Lancet Gastroenterol. Hepatol..

[5] Rinella M.E., Neuschwander-Tetri B.A., Siddiqui M.S., Abdelmalek M.F., Caldwell S., Barb D., Kleiner D.E., Loomba R. (2023). AASLD Practice Guidance on the Clinical Assessment and Management of Nonalcoholic Fatty Liver Disease. Hepatology.

[6] Dai W., Ye L., Liu A., Wen S.W., Deng J., Wu X., Lai Z. (2017). Prevalence of Nonalcoholic Fatty Liver Disease in Patients with Type 2 Diabetes Mellitus: A Meta-Analysis. Medicine.

[7] Tacke F., Horn P., Wong V.W.-S., Ratziu V., Bugianesi E., Francque S., Zelber-Sagi S., Valenti L., Roden M., Schick F. (2024). EASL–EASD–EASO Clinical Practice Guidelines on the Management of Metabolic Dysfunction-Associated Steatotic Liver Disease (MASLD). J. Hepatol..

[8] Berardo C., Di Pasqua L.G., Cagna M., Richelmi P., Vairetti M., Ferrigno A. (2020). Nonalcoholic Fatty Liver Disease and Non-Alcoholic Steatohepatitis: Current Issues and Future Perspectives in Preclinical and Clinical Research. Int. J. Mol. Sci..

[9] Hughes J.W., Levin J.B., Sajatovic M., Sclair S.N. (2025). Semaglutide for Metabolic Dysfunction-Associated Steatohepatitis (MASH): Estimating Eligibility from the 2021-2023 National Health and Nutrition Examination Survey (NHANES). Hepatol. Commun..

[10] Ferrigno A., Irene L., Campagnoli M., Barbieri A., Marchesi N., Pascale A., Croce A.C., Vairetti M., Giuseppina L., Pasqua D. (2023). MCD Diet Modulates HuR and Oxidative Stress-Related HuR Targets in Rats. Int. J. Mol. Sci..

[11] Serviddio G., Bellanti F., Vendemiale G., Altomare E. (2011). Mitochondrial Dysfunction in Nonalcoholic Steatohepatitis. Expert Rev. Gastroenterol. Hepatol..

[12] Begriche K., Massart J., Robin M.A., Bonnet F., Fromenty B. (2013). Mitochondrial Adaptations and Dysfunctions in Nonalcoholic Fatty Liver Disease. Hepatology.

[13] Pacana T., Sanyal A.J. (2015). Recent Advances in Understanding/Management of Non-Alcoholic Steatohepatitis. F1000Prime Rep..

[14] Prasun P., Ginevic I., Oishi K. (2021). Mitochondrial Dysfunction in Nonalcoholic Fatty Liver Disease and Alcohol Related Liver Disease. Transl. Gastroenterol. Hepatol..

[15] García-Ruiz C., Fernández-Checa J.C. (2018). Mitochondrial Oxidative Stress and Antioxidants Balance in Fatty Liver Disease. Hepatol. Commun..

[16] Hodson L., McQuaid S.E., Humphreys S.M., Milne R., Fielding B.A., Frayn K.N., Karpe F. (2010). Greater Dietary Fat Oxidation in Obese Compared with Lean Men: An Adaptive Mechanism to Prevent Liver Fat Accumulation?. Am. J. Physiol. Endocrinol. Metab..

[17] Sunny N.E., Parks E.J., Browning J.D., Burgess S.C. (2011). Excessive Hepatic Mitochondrial TCA Cycle and Gluconeogenesis in Humans with Nonalcoholic Fatty Liver Disease. Cell Metab..

[18] Quinlan C.L., Perevoshchikova I.V., Hey-Mogensen M., Orr A.L., Brand M.D. (2013). Sites of Reactive Oxygen Species Generation by Mitochondria Oxidizing Different Substrates. Redox Biol..

[19] Fromenty B., Roden M. (2023). Mitochondrial Alterations in Fatty Liver Diseases. J. Hepatol..

[20] Liu Q., Zhang D., Hu D., Zhou X., Zhou Y. (2018). The Role of Mitochondria in NLRP3 Inflammasome Activation. Mol. Immunol..

[21] Rolo A.P., Teodoro J.S., Palmeira C.M. (2012). Role of Oxidative Stress in the Pathogenesis of Nonalcoholic Steatohepatitis. Free Radic. Biol. Med..

[22] Auger C., Alhasawi A., Contavadoo M., Appanna V.D. (2015). Dysfunctional Mitochondrial Bioenergetics and the Pathogenesis of Hepatic Disorders. Front. Cell Dev. Biol..

[23] Murphy M.P. (2009). How Mitochondria Produce Reactive Oxygen Species. Biochem. J..

[24] Janssen R.J.R.J., Nijtmans L.G., van den Heuvel L.P., Smeitink J.A.M. (2006). Mitochondrial Complex I: Structure, Function and Pathology. J. Inherit. Metab. Dis..

[25] Abdelmegeed M.A., Song B.-J. (2014). Functional Roles of Protein Nitration in Acute and Chronic Liver Diseases. Oxid. Med. Cell. Longev..

[26] Cameron A.R., Logie L., Patel K., Erhardt S., Bacon S., Middleton P., Harthill J., Forteath C., Coats J.T., Kerr C. (2018). Metformin Selectively Targets Redox Control of Complex I Energy Transduction. Redox Biol..

[27] LaMoia T.E., Shulman G.I. (2021). Cellular and Molecular Mechanisms of Metformin Action. Endocr. Rev..

[28] Savage D.B., Choi C.S., Samuel V.T., Liu Z.-X., Zhang D., Wang A., Zhang X.-M., Cline G.W., Yu X.X., Geisler J.G. (2006). Reversal of Diet-Induced Hepatic Steatosis and Hepatic Insulin Resistance by Antisense Oligonucleotide Inhibitors of Acetyl-CoA Carboxylases 1 and 2. J. Clin. Investig..

[29] Mastrototaro L., Roden M. (2021). Insulin Resistance and Insulin Sensitizing Agents. Metabolism.

[30] Pharaoh G., Ostrom E.L., Stuppard R., Campbell M., Borghardt J.M., Franti M., Filareto A., Marcinek D.J. (2023). A Novel Mitochondrial Complex I ROS Inhibitor Partially Improves Muscle Regeneration in Adult but not Old Mice. Redox Biol..

[31] Di Pasqua L.G., Berardo C., Cagna M., Mannucci B., Milanesi G., Croce A.C., Ferrigno A., Vairetti M. (2021). Long-Term Cold Storage Preservation Does Not Affect Fatty Livers from Rats Fed with a Methionine and Choline Deficient Diet. Lipids Health Dis..

[32] Esterbauer H., Cheeseman K.H. (1990). Determination of Aldehydic Lipid Peroxidation Products: Malonaldehyde and 4-Hydroxynonenal. Methods Enzymol..

[33] Croce A.C., Ferrigno A., Di Pasqua L.G., Berardo C., Piccolini V.M., Bertone V., Bottiroli G., Vairetti M. (2016). Autofluorescence Discrimination of Metabolic Fingerprint in Nutritional and Genetic Fatty Liver Models. J. Photochem. Photobiol. B Biol..

[34] Di Pasqua L.G., Berardo C., Rizzo V., Richelmi P., Croce A.C., Vairetti M., Ferrigno A. (2016). MCD Diet-Induced Steatohepatitis is Associated with Alterations in Asymmetric Dimethylarginine (ADMA) and Its Transporters. Mol. Cell. Biochem..

[35] Lehninger A.L., Vercesi A., Bababunmi E.A. (1978). Regulation of Ca^2+^ Release from Mitochondria by the Oxidation-Reduction State of Pyridine Nucleotides. Proc. Natl. Acad. Sci. USA.

[36] Lowry O.H., Rosebrough N.J., Farr A.L., Randall R.J. (1951). Protein Measurement with the Folin Phenol Reagent. J. Biol. Chem..

[37] Ferrigno A., Berardo C., Di Pasqua L.G., Siciliano V., Richelmi P., Nicoletti F., Vairetti M. (2018). Selective Blockade of the Metabotropic Glutamate Receptor mGluR5 Protects Mouse Livers in in Vitro and Ex Vivo Models of Ischemia Reperfusion Injury. Int. J. Mol. Sci..

[38] Croce A.C., Ferrigno A., Di Pasqua L.G., Berardo C., Bottiroli G., Vairetti M. (2017). NAD(P)H and Flavin Autofluorescence Correlation with ATP in Rat Livers with Different Metabolic Steady-State Conditions. Photochem. Photobiol..

[39] Lyn-Cook L.E., Lawton M., Tong M., Silbermann E., Longato L., Jiao P., Mark P., Wands J.R., Xu H., de la Monte S.M. (2009). Hepatic Ceramide May Mediate Brain Insulin Resistance and Neurodegeneration in Type 2 Diabetes and Non-Alcoholic Steatohepatitis. J. Alzheimers Dis..

[40] Ferrigno A., Cagna M., Bosco O., Trucchi M., Berardo C., Nicoletti F., Vairetti M., Di Pasqua L.G. (2023). MPEP Attenuates Intrahepatic Fat Accumulation in Obese Mice. Int. J. Mol. Sci..

[41] Turato C., Vairetti M., Cagna M., Biasiolo A., Ferrigno A., Quarta S., Ruvoletto M., De Siervi S., Pontisso P., Di Pasqua L.G. (2022). SerpinB3 Administration Protects Liver against Ischemia-Reperfusion Injury. Eur. J. Histochem..

[42] Chomczynski P., Mackey K. (1995). Substitution of Chloroform by Bromo-Chloropropane in the Single-Step Method of RNA Isolation. Anal. Biochem..

[43] Bustin S.A., Benes V., Garson J.A., Hellemans J., Huggett J., Kubista M., Mueller R., Nolan T., Pfaffl M.W., Shipley G.L. (2009). The MIQE Guidelines: Minimum Information for Publication of Quantitative Real-Time PCR Experiments. Clin. Chem..

[44] Di Pasqua L.G., Cagna M., Palladini G., Croce A.C., Cadamuro M., Fabris L., Perlini S., Adorini L., Ferrigno A., Vairetti M. (2024). FXR Agonists INT-787 and OCA Increase RECK and Inhibit Liver Steatosis and Inflammation in Diet-Induced Ob/Ob Mouse Model of NASH. Liver Int..

[45] Tsikas D. (2017). Assessment of Lipid Peroxidation by Measuring Malondialdehyde (MDA) and Relatives in Biological Samples: Analytical and Biological Challenges. Anal. Biochem..

[46] Silva A.M., Oliveira P.J. (2012). Evaluation of Respiration with Clark Type Electrode in Isolated Mitochondria and Permeabilized Animal Cells. Mitochondrial Bioenergetics.

[47] Salmon J.M., Kohen E., Viallet P., Hirschberg J.G., Wouters A.W., Kohen C., Thorell B. (1982). Microspectrofluorometric Approach to the Study of Free/Bound NAD(P)H Ratio as Metabolic Indicator in Various Cell Types. Photochem. Photobiol..

[48] Nakamura M., Bhatnagar A., Sadoshima J. (2012). Overview of Pyridine Nucleotides Review Series. Circ. Res..

[49] Croce A.C., Bottiroli G. (2014). Autofluorescence Spectroscopy and Imaging: A Tool for Biomedical Research and Diagnosis. Eur. J. Histochem..

[50] Dong J., Chen L., Ye F., Tang J., Liu B., Lin J., Zhou P.-H., Lu B., Wu M., Lu J.-H. (2024). Mic19 Depletion Impairs Endoplasmic Reticulum-Mitochondrial Contacts and Mitochondrial Lipid Metabolism and Triggers Liver Disease. Nat. Commun..

[51] Ikon N., Ryan R.O. (2017). Cardiolipin and Mitochondrial Cristae Organization. Biochim. Biophys. Acta Biomembr..

[52] Li J., Romestaing C., Han X., Li Y., Hao X., Wu Y., Sun C., Liu X., Jefferson L.S., Xiong J. (2010). Cardiolipin Remodeling by ALCAT1 Links Oxidative Stress and Mitochondrial Dysfunction to Obesity. Cell Metab..

[53] Gong F., Gao L., Ding T. (2019). IDH2 Protects against Nonalcoholic Steatohepatitis by Alleviating Dyslipidemia Regulated by Oxidative Stress. Biochem. Biophys. Res. Commun..

[54] Han S.J., Choi H.S., Kim J.I., Park J.-W., Park K.M. (2018). IDH2 Deficiency Increases the Liver Susceptibility to Ischemia-Reperfusion Injury via Increased Mitochondrial Oxidative Injury. Redox Biol..

[55] Vairetti M., Di Pasqua L.G., Cagna M., Richelmi P., Ferrigno A., Berardo C. (2021). Changes in Glutathione Content in Liver Diseases: An Update. Antioxidants.

[56] Cadenas S. (2018). Mitochondrial Uncoupling, ROS Generation and Cardioprotection. Biochim. Biophys. Acta Bioenerg..

[57] Vallejo F.A., Vanni S., Graham R.M. (2021). UCP2 as a Potential Biomarker for Adjunctive Metabolic Therapies in Tumor Management. Front. Oncol..

[58] Hao L., Li S., Chen G., Hu X. (2024). Regulation of UCP2 in Nonalcoholic Fatty Liver Disease: From Mechanisms to Natural Product. Chem. Biol. Drug Des..

[59] Chambel S.S., Santos-Gonçalves A., Duarte T.L. (2015). The Dual Role of Nrf2 in Nonalcoholic Fatty Liver Disease: Regulation of Antioxidant Defenses and Hepatic Lipid Metabolism. BioMed Res. Int..

[60] Kang Y., Song Y., Luo Y., Song J., Li C., Yang S., Guo J., Yu J., Zhang X. (2022). Exosomes Derived from Human Umbilical Cord Mesenchymal Stem Cells Ameliorate Experimental Non-Alcoholic Steatohepatitis via Nrf2/NQO-1 Pathway. Free Radic. Biol. Med..

[61] Fang C., Pan J., Qu N., Lei Y., Han J., Zhang J., Han D. (2022). The AMPK Pathway in Fatty Liver Disease. Front. Physiol..

[62] Feng J., Qiu S., Zhou S., Tan Y., Bai Y., Cao H., Guo J., Su Z. (2022). mTOR: A Potential New Target in Nonalcoholic Fatty Liver Disease. Int. J. Mol. Sci..

[63] Zeng J., Zhu B., Su M. (2018). Autophagy Is Involved in Acetylshikonin Ameliorating Non-Alcoholic Steatohepatitis through AMPK/mTOR Pathway. Biochem. Biophys. Res. Commun..

[64] Farrell G.C., van Rooyen D., Gan L., Chitturi S. (2012). NASH Is an Inflammatory Disorder: Pathogenic, Prognostic and Therapeutic Implications. Gut Liver.

[65] Popko K., Gorska E., Stelmaszczyk-Emmel A., Plywaczewski R., Stoklosa A., Gorecka D., Pyrzak B., Demkow U. (2010). Proinflammatory Cytokines IL-6 and TNF-α and the Development of Inflammation in Obese Subjects. Eur. J. Med. Res..

[66] Duarte S., Baber J., Fujii T., Coito A.J. (2015). Matrix Metalloproteinases in Liver Injury, Repair and Fibrosis. Matrix Biol..

[67] Palladini G., Di Pasqua L.G., Croce A.C., Ferrigno A., Vairetti M. (2023). Recent Updates on the Therapeutic Prospects of Reversion-Inducing Cysteine-Rich Protein with Kazal Motifs (RECK) in Liver Injuries. Int. J. Mol. Sci..

[68] Jorgačević B., Mladenović D., Ninković M., Prokić V., Stanković M.N., Aleksić V., Cerović I., Vukićević R.J., Vučević D., Stanković M. (2014). Dynamics of Oxidative/Nitrosative Stress in Mice with Methionine-Choline-Deficient Diet-Induced Nonalcoholic Fatty Liver Disease. Hum. Exp. Toxicol..

[69] Parihar M.S., Nazarewicz R.R., Kincaid E., Bringold U., Ghafourifar P. (2008). Association of Mitochondrial Nitric Oxide Synthase Activity with Respiratory Chain Complex I. Biochem. Biophys. Res. Commun..

[70] Ross D., Siegel D. (2021). The Diverse Functionality of NQO1 and Its Roles in Redox Control. Redox Biol..

[71] Pospisilik J.A., Knauf C., Joza N., Benit P., Orthofer M., Cani P.D., Ebersberger I., Nakashima T., Sarao R., Neely G. (2007). Targeted Deletion of AIF Decreases Mitochondrial Oxidative Phosphorylation and Protects from Obesity and Diabetes. Cell.

[72] Moser M.A., Chun O.K. (2016). Vitamin C and Heart Health: A Review Based on Findings from Epidemiologic Studies. Int. J. Mol. Sci..

[73] Raza S., Tewari A., Rajak S., Sinha R.A. (2021). Vitamins and Non-Alcoholic Fatty Liver Disease: A Molecular Insight. Liver Res..

[74] Fernandez-Marcos P.J., Nóbrega-Pereira S. (2016). NADPH: New Oxygen for the ROS Theory of Aging. Oncotarget.

[75] Caballero F., Fernández A., Matías N., Martínez L., Fucho R., Elena M., Caballeria J., Morales A., Fernández-Checa J.C., García-Ruiz C. (2010). Specific Contribution of Methionine and Choline in Nutritional Nonalcoholic Steatohepatitis: Impact on Mitochondrial S-Adenosyl-L-Methionine and Glutathione. J. Biol. Chem..

[76] Stephenson K., Kennedy L., Hargrove L., Demieville J., Thomson J., Alpini G., Francis H. (2018). Updates on Dietary Models of Nonalcoholic Fatty Liver Disease: Current Studies and Insights. Gene Expr..

[77] Herzig S., Shaw R.J. (2018). AMPK: Guardian of Metabolism and Mitochondrial Homeostasis. Nat. Rev. Mol. Cell Biol..

[78] Marcondes-de-Castro I.A., Reis-Barbosa P.H., Marinho T.S., Aguila M.B., Mandarim-de-Lacerda C.A. (2023). AMPK/mTOR Pathway Significance in Healthy Liver and Non-Alcoholic Fatty Liver Disease and Its Progression. J. Gastroenterol. Hepatol..

[79] Szwed A., Kim E., Jacinto E. (2021). Regulation and Metabolic Functions of mTORC1 and mTORC2. Physiol. Rev..

[80] Smith B.K., Marcinko K., Desjardins E.M., Lally J.S., Ford R.J., Steinberg G.R. (2016). Treatment of Nonalcoholic Fatty Liver Disease: Role of AMPK. Am. J. Physiol.-Endocrinol. Metab..

[81] Esquejo R.M., Salatto C.T., Delmore J., Albuquerque B., Reyes A., Shi Y., Moccia R., Cokorinos E., Peloquin M., Monetti M. (2018). Activation of Liver AMPK with PF-06409577 Corrects NAFLD and Lowers Cholesterol in Rodent and Primate Preclinical Models. eBioMedicine.

[82] Garcia D., Hellberg K., Chaix A., Wallace M., Herzig S., Badur M.G., Lin T., Shokhirev M.N., Pinto A.F.M., Ross D.S. (2019). Genetic Liver-Specific AMPK Activation Protects against Diet-Induced Obesity and NAFLD. Cell Rep..

[83] Ge X., Wang C., Chen H., Liu T., Chen L., Huang Y., Zeng F., Liu B. (2020). Luteolin Cooperated with Metformin Hydrochloride Alleviates Lipid Metabolism Disorders and Optimizes Intestinal Flora Compositions of High-Fat Diet Mice. Food Funct..

[84] Zhou G., Myers R., Li Y., Chen Y., Shen X., Fenyk-Melody J., Wu M., Ventre J., Doebber T., Fujii N. (2001). Role of AMP-Activated Protein Kinase in Mechanism of Metformin Action. J. Clin. Investig..

[85] Boudaba N., Marion A., Huet C., Pierre R., Viollet B., Foretz M. (2018). AMPK Re-Activation Suppresses Hepatic Steatosis but Its Downregulation Does Not Promote Fatty Liver Development. eBioMedicine.

[86] Koyama Y., Brenner D.A. (2017). Liver Inflammation and Fibrosis. J. Clin. Investig..

[87] Santhekadur P.K., Kumar D.P., Sanyal A.J. (2018). Preclinical Models of Non-Alcoholic Fatty Liver Disease. J. Hepatol..

[88] Rahman S.M., Schroeder-Gloeckler J.M., Janssen R.C., Jiang H., Qadri I., Maclean K.N., Friedman J.E. (2007). CCAAT/Enhancing Binding Protein β Deletion in Mice Attenuates Inflammation, Endoplasmic Reticulum Stress, and Lipid Accumulation in Diet-Induced Nonalcoholic Steatohepatitis. Hepatology.

[89] Gloire G., Piette J. (2009). Redox Regulation of Nuclear Post-Translational Modifications During NF-κB Activation. Antioxid. Redox Signal..

[90] Salamone F., Galvano F., Cappello F., Mangiameli A., Barbagallo I., Li Volti G. (2012). Silibinin Modulates Lipid Homeostasis and Inhibits Nuclear Factor Kappa B Activation in Experimental Nonalcoholic Steatohepatitis. Transl. Res..

[91] Feldstein A.E., Werneburg N.W., Li Z., Bronk S.F., Gores G.J. (2006). Bax Inhibition Protects against Free Fatty Acid-Induced Lysosomal Permeabilization. Am. J. Physiol. Gastrointest. Liver Physiol..

[92] Malhi H., Gores G.J. (2008). Molecular Mechanisms of Lipotoxicity in Nonalcoholic Fatty Liver Disease. Semin. Liver Dis..

[93] Elsharkawy A.M., Mann D.A. (2007). Nuclear Factor-kappaB and the Hepatic Inflammation-Fibrosis-Cancer Axis. Hepatology.

[94] Zhang Y., Ren L., Tian Y., Guo X., Wei F., Zhang Y. (2024). Signaling Pathways That Activate Hepatic Stellate Cells during Liver Fibrosis. Front. Med..

[95] Gao J., Liu Q., Li J., Hu C., Zhao W., Ma W., Yao M., Xing L. (2020). Fibroblast Growth Factor 21 Dependent TLR4/MYD88/NF-κB Signaling Activation Is Involved in Lipopolysaccharide-Induced Acute Lung Injury. Int. Immunopharmacol..

[96] Knorr J., Wree A., Tacke F., Feldstein A.E. (2020). The NLRP3 Inflammasome in Alcoholic and Nonalcoholic Steatohepatitis. Semin. Liver Dis..

[97] Ramos-Tovar E., Muriel P. (2020). Molecular Mechanisms That Link Oxidative Stress, Inflammation, and Fibrosis in the Liver. Antioxidants.

[98] Hu L., Su L., Dong Z., Wu Y., Lv Y., George J., Wang J. (2019). AMPK Agonist AICAR Ameliorates Portal Hypertension and Liver Cirrhosis via NO Pathway in the BDL Rat Model. J. Mol. Med..

[99] Palladini G., Di Pasqua L.G., Berardo C., Siciliano V., Richelmi P., Perlini S., Ferrigno A., Vairetti M. (2019). Animal Models of Steatosis (NAFLD) and Steatohepatitis (NASH) Exhibit Hepatic Lobe-Specific Gelatinases Activity and Oxidative Stress. Can. J. Gastroenterol. Hepatol..

[100] Almishri W., Swain L.A., D’Mello C., Le T.S., Urbanski S.J., Nguyen H.H. (2022). ADAM Metalloproteinase Domain 17 Regulates Cholestasis-Associated Liver Injury and Sickness Behavior Development in Mice. Front. Immunol..

[101] Schmidt-Arras D., Rose-John S. (2019). Regulation of Fibrotic Processes in the Liver by ADAM Proteases. Cells.

[102] Liao S., Lin Y., Liu L., Yang S., Lin Y., He J., Shao Y. (2023). ADAM10-a “Multitasker” in Sepsis: Focus on Its Posttranslational Target. Inflamm. Res..

[103] Zhu C., Kim K., Wang X., Bartolome A., Salomao M., Dongiovanni P., Meroni M., Graham M.J., Yates K.P., Diehl A.M. (2018). Hepatocyte Notch Activation Induces Liver Fibrosis in Nonalcoholic Steatohepatitis. Sci. Transl. Med..

[104] Yong S.-B., Song Y., Kim Y.-H. (2017). Visceral Adipose Tissue Macrophage-Targeted TACE Silencing to Treat Obesity-Induced Type 2 Diabetes. Biomaterials.

[105] Serino M., Menghini R., Fiorentino L., Amoruso R., Mauriello A., Lauro D., Sbraccia P., Hribal M.L., Lauro R., Federici M. (2007). Mice Heterozygous for Tumor Necrosis Factor-Alpha Converting Enzyme Are Protected from Obesity-Induced Insulin Resistance and Diabetes. Diabetes.

[106] Bourd-Boittin K., Basset L., Bonnier D., L’helgoualc’h A., Samson M., Théret N. (2009). CX3CL1/Fractalkine Shedding by Human Hepatic Stellate Cells: Contribution to Chronic Inflammation in the Liver. J. Cell. Mol. Med..

[107] Wang K., Lin B., Brems J.J., Gamelli R.L. (2013). Hepatic Apoptosis Can Modulate Liver Fibrosis through TIMP1 Pathway. Apoptosis.

[108] Li X., Peng J., Sun Z., Tian H., Duan X., Liu L., Ma X., Feng Q., Liu P., Hu Y. (2016). Chinese Medicine CGA Formula Ameliorates DMN-Induced Liver Fibrosis in Rats via Inhibiting MMP2/9, TIMP1/2 and the TGF-β/Smad Signaling Pathways. Acta Pharmacol. Sin..

[109] Wu L., Zhang Q., Mo W., Feng J., Li S., Li J., Liu T., Xu S., Wang W., Lu X. (2017). Quercetin Prevents Hepatic Fibrosis by Inhibiting Hepatic Stellate Cell Activation and Reducing Autophagy via the TGF-β1/Smads and PI3K/Akt Pathways. Sci. Rep..

[110] Busk T.M., Bendtsen F., Nielsen H.J., Jensen V., Brünner N., Møller S. (2014). TIMP-1 in Patients with Cirrhosis: Relation to Liver Dysfunction, Portal Hypertension, and Hemodynamic Changes. Scand. J. Gastroenterol..

[111] Cunningham R., Sheldon R., Meers G., Kandikattu H.K., Chandrasekar B., Rector R.S. (2017). Western Diet Feeding Downregulates Hepatic RECK Expression and Induces NASH with Fibrosis. FASEB J..

[112] Peng X., Wu W., Zhu B., Sun Z., Ji L., Ruan Y., Zhou M., Zhou L., Gu J. (2014). Activation of Farnesoid X Receptor Induces RECK Expression in Mouse Liver. Biochem. Biophys. Res. Commun..

[113] Palladini G., Di Pasqua L.G., Cagna M., Croce A.C., Perlini S., Mannucci B., Profumo A., Ferrigno A., Vairetti M. (2022). MCD Diet Rat Model Induces Alterations in Zinc and Iron during NAFLD Progression from Steatosis to Steatohepatitis. Int. J. Mol. Sci..

